# Developing photoreceptor-based models of visual attraction in riverine tsetse, for use in the engineering of more-attractive polyester fabrics for control devices

**DOI:** 10.1371/journal.pntd.0005448

**Published:** 2017-03-17

**Authors:** Roger D. Santer

**Affiliations:** Institute of Biological, Environmental, and Rural Sciences, Aberystwyth University, Aberystwyth, Ceredigion, SY23 3FG United Kingdom; Liverpool School of Tropical Medicine, UNITED KINGDOM

## Abstract

Riverine tsetse transmit the parasites that cause the most prevalent form of human African trypanosomiasis, Gambian HAT. In response to the imperative for cheap and efficient tsetse control, insecticide-treated ‘tiny targets’ have been developed through refinement of tsetse attractants based on blue fabric panels. However, modern blue polyesters used for this purpose attract many less tsetse than traditional phthalogen blue cottons. Therefore, colour engineering polyesters for improved attractiveness has great potential for tiny target development. Because flies have markedly different photoreceptor spectral sensitivities from humans, and the responses of these photoreceptors provide the inputs to their visually guided behaviours, it is essential that polyester colour engineering be guided by fly photoreceptor-based explanations of tsetse attraction. To this end, tsetse attraction to differently coloured fabrics was recently modelled using the calculated excitations elicited in a generic set of fly photoreceptors as predictors. However, electrophysiological data from tsetse indicate the potential for modified spectral sensitivities versus the generic pattern, and processing of fly photoreceptor responses within segregated achromatic and chromatic channels has long been hypothesised. Thus, I constructed photoreceptor-based models explaining the attraction of *G*. *f*. *fuscipes* to differently coloured tiny targets recorded in a previously published investigation, under differing assumptions about tsetse spectral sensitivities and organisation of visual processing. Models separating photoreceptor responses into achromatic and chromatic channels explained attraction better than earlier models combining weighted photoreceptor responses in a single mechanism, regardless of the spectral sensitivities assumed. However, common principles for fabric colour engineering were evident across the complete set of models examined, and were consistent with earlier work. Tools for the calculation of fly photoreceptor excitations are available with this paper, and the ways in which these and photoreceptor-based models of attraction can provide colorimetric values for the engineering of more-attractively coloured polyester fabrics are discussed.

## Author summary

Tsetse flies transmit the parasites that cause sleeping sickness. Tsetse control can contribute to disease control thanks to cheap and efficient ‘tiny targets’ that attract tsetse using a panel of blue fabric, a highly attractive colour for the flies. However, the modern blue polyesters employed are only about half as attractive as traditional phthalogen blue cottons. It will be possible to engineer more-attractive polyesters using techniques based on those already employed for fabric colour matching to the human eye. However, because fly photoreceptors differ to those of humans, these methods must be modified to evaluate colour from the fly’s eye view. This paper continues recent work attempting to explain tsetse attraction to differently coloured fabrics using the calculated responses of fly photoreceptors to those fabrics. In particular, this paper investigates several different assumptions about the sensitivities of tsetse photoreceptors and the ways in which their responses are processed. Regardless of these assumptions, common principles for the engineering of attractive fabrics were determined. The tools provided with this paper, along with fabric engineering methods already in use, will permit the engineering of more-attractively coloured polyesters for the increased efficiency of tsetse and sleeping sickness control.

## Introduction

Tsetse flies (*Glossina* spp.) are blood-feeding flies of sub-Saharan Africa, and their bites transmit the trypanosome parasites that cause sleeping sickness in humans (human African trypanosomiasis, HAT), and nagana in cattle (animal African trypanosomiasis, AAT) [1]. There are no vaccines or chemoprophylaxes to prevent HAT, and the diagnostics and treatments currently available are imperfect [2]. Therefore, tsetse control can provide an important component of disease control [3,4,5]. However, to do so, it is imperative that the cost and efficacy of tsetse control are optimised.

### The need for efficient control devices for riverine tsetse

There are two forms of HAT, each caused by a different subspecies of *Trypanosoma brucei*. A small minority of cases (ca. 2%) comprise an acute disease termed Rhodesian HAT that occurs in Eastern and Southern Africa [1]. These cases are caused by *T*. *b*. *rhodesiense* for which savannah, or Morsitans species group, tsetse are the most important vectors. Because Rhodesian HAT is a zoonosis, tsetse control is central to disease control [6]. Large, insecticide-treated blue and/or black cloth panels with accompanying odour lures, and insecticide-treated cattle, have both proved effective in controlling savannah tsetse [7,8]. However, the vast majority of HAT cases (ca. 98%) comprise a chronic disease termed Gambian HAT that occurs in Central and Western Africa [1]. This form of the disease is caused by *T*. *b*. *gambiense* and chiefly transmitted by riverine, or Palpalis species group, tsetse. Of these, *G*. *fuscipes* spp. are estimated to transmit 90% of all Gambian HAT [3,5]. Unlike Rhodesian HAT, Gambian HAT is considered an anthroponosis, and case detection and treatment programmes are the predominant method of disease control. This is because control methods developed for savannah tsetse are not cost effective or logistically feasible for the control of riverine tsetse in remote, rural locations, and where cattle rearing densities are low [3,4,6]. However, case detection and treatment programmes suffer from diagnostic insensitivity and incomplete attendance of the local population for screening, causing under-detection and allowing disease transmission to be sustained [9]. Tsetse control, meanwhile, has proved effective in reducing tsetse numbers below those required for HAT transmission, and sustaining such a programme, perhaps in concert with active case detection and treatment, might achieve local elimination of the disease [4,5]. Therefore, concerted efforts have been underway to improve the cost and efficiency of control devices for riverine tsetse so that they might contribute to efforts to eliminate Gambian HAT (e.g. [10,11,12,13,14,15,16]).

Pronounced differences are evident in the behaviour of riverine and savannah tsetse, with the former less repelled by visual and olfactory stimuli emanating from humans, more likely to feed from smaller hosts such as reptiles and small mammals, and generally less responsive to odour lures [7]. In addition, although larger targets are most attractive to both riverine and savannah tsetse, the former are relatively more attracted to smaller visual targets than the latter [7,12]. These behavioural differences are probably not the result of differences in tsetse physiology. Instead, narrow and densely vegetated riverine habitats mean that odour plumes are of limited utility in host seeking, and the reduced probability of encountering hosts in such habitats necessitates less selectivity when one is encountered [7,17]. Identification of these crucial behavioural differences has allowed the development of ‘tiny targets’ for riverine tsetse, which comprise a 0.25 m x 0.25 m blue polyester panel adjacent to a 0.25 m x 0.25 m black polyethylene mosquito net panel, with both panels impregnated with deltamethrin insecticide [5,10,12,14]. The blue fabric panel serves to attract tsetse, whilst the mosquito net panel is thought to intercept circling flies, overcoming their reduced tendency to alight directly on small visual targets [12]. Versus the large devices used for savannah tsetse, tiny targets offer considerable savings in costs associated with materials, transport, and deployment, but with only a small penalty in terms of reduced attractiveness to tsetse [10,12]. As a result of these improvements, tsetse control can now form an important component of Gambian HAT control [4,5,16]. As an added benefit, such cheap and efficient technology might permit community-led control operations, and through them some protection against disease resurgences that have resulted from the neglect of control operations at times of political instability (e.g. [2]).

### The potential for colour optimisation to improve tsetse control devices

Tiny targets are undoubtedly a hugely important innovation that already permits cost effective tsetse control, but their efficiency might be improved still further through optimisation of the colour of the attractive polyester panel. Multiple large scale field studies pre-dating the development of tiny targets established that colour was an important determinant of tsetse attraction, and that phthalogen blue-dyed cotton was highly attractive to tsetse [8,14,18,19]. Phthalogen blue cotton is now reportedly difficult to obtain, whilst polyester is the material of choice for tiny targets because it is lighter, more robust under field conditions, and holds insecticide more effectively [14]. Although the blue polyester used in tsetse control devices is sometimes referred to as ‘phthalogen blue’ polyester, phthalogen blue dye can only be applied to cotton, and the reflectance spectra of such polyester fabrics differ subtly from that of the original cotton material [14,20]. Experiments conducted during the development of tiny targets found that identically sized panels of ‘phthalogen blue’ polyester frequently attracted significantly less tsetse than phthalogen blue cotton, and the numbers of tsetse caught at these targets was often only ca. 50% of those caught at a phthalogen blue cotton standard [14]. The implication of this is clear: identification of polyester fabrics that achieve the same level of efficiency as phthalogen blue cotton has the potential to as much as double the efficiency of tiny targets. Although such a polyester fabric could not be identified through screening a wide selection of differently coloured polyesters in field trials [14], I argue that an attractively coloured fabric can be deliberately engineered if the mechanistic basis of tsetse attraction is understood. On that basis, polyester dye concentrations and combinations that would best exploit the implicated mechanism can be identified, using techniques based on those already employed to colour engineer fabrics for the human eye [21,22,23].

**Fig 1:**
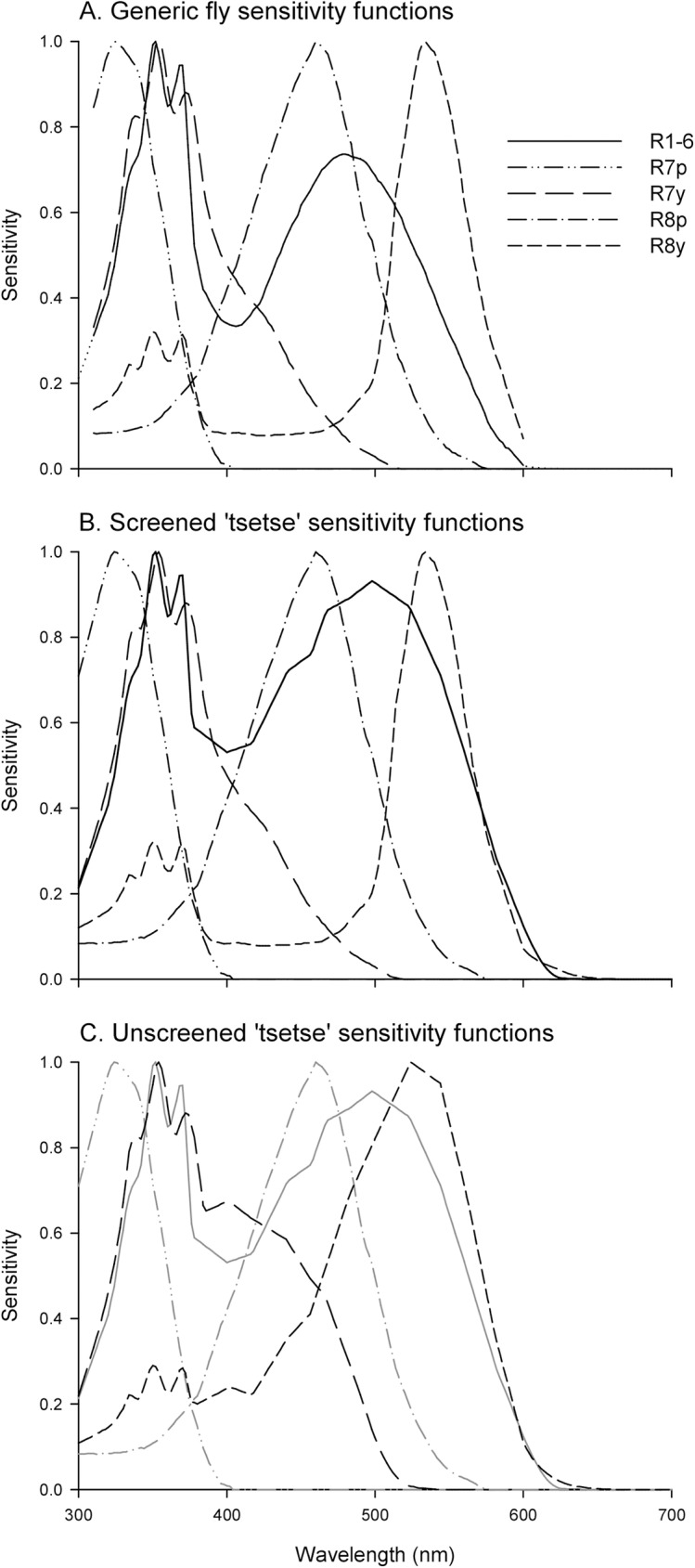
Fly photoreceptor sensitivity functions. (A) Generic fly sensitivity functions typical of *Musca* and *Calliphora* based upon [26]. The functions are plotted between 310 and 600 nm as they were applied in earlier work on tsetse attraction [21,22]. Dotted lines extrapolate the R1-6 sensitivity function for use in the current study. (B) Sensitivity functions used in the current analysis to represent the hypothesised spectral sensitivities of a tsetse fly with screening from carotenoid pigments equivalent to that observed in *Musca* and *Calliphora*. The R7-8 sensitivity functions are based on those in panel A, and the R1-6 sensitivity function has been constructed from electrophysiological data for *G*. *m*. *morsitans* [29]. Note the sensitivity peak close to 500 nm and approximately 10 nm longer than that in panel A. (C) Sensitivity functions used in the current analysis to represent the spectral sensitivities of a tsetse fly lacking in carotenoid screening pigments as a result of dietary deprivation. R1-6, R7p, and R8p sensitivity functions are as in panel B, and broader spectral sensitivity functions have been constructed for R7y and R8y based upon data from *G*. *m*. *morsitans* [29]. The elevated sensitivity of these photoreceptors in the blue region of the spectrum results from the absence of carotenoid screening pigments in the R7y rhabdoms [29].

Visual information is received by an animal’s photoreceptors, and it is the responses of these photoreceptors that provide the inputs to visually guided behaviour and the basis of colour perceptions [23,24,25]. For this reason, colorimetric approaches to match colours for the human eye do not attempt to match the desired reflectance spectrum, but instead design a reflectance spectrum that evokes the same response in the human cone photoreceptors [23]. Since flies differ from humans in the number and spectral sensitivity of their photoreceptors, human colour perceptions are not useful in understanding the visual behaviour of flies. Fortunately, photoreceptor spectral sensitivities are well established for *Musca* and *Calliphora* spp., and are considered typical of all higher flies [26,27,28]. Methods to model photoreceptor responses to measured reflectance spectra using such sensitivity functions are now well established [24,25]. Each ommatidium in the dipteran compound eye contains eight photoreceptors (also called retinula cells), named R1-R8. R1-6 are broadband photoreceptors that are similar across all ommatidia in the eye, and they make output synapses in the lamina of the optic lobe [26,28] (see Fig 1). Photoreceptors R7 and R8 are stacked centrally within each ommatidium and bypass the lamina to make output synapses in the medulla of the optic lobe. Excluding specialised areas of the eye such as that for the detection of polarised light, R7 and R8 each occur in two forms [26,28,29]. In 70% of ommatidia the ‘y’ (yellow) form occurs, where C_40_ carotenoid screening pigments are present in the R7 rhabdoms and these shape the sensitivity of both R7y and R8y receptors [26,28,30,31] (Fig 1A). In the remaining 30% of ommatidia, the ‘p’ (pale) form of these photoreceptors occurs [26,28,30,31] (Fig 1A). Based on these spectral sensitivities and field data from previously published studies of savannah and riverine tsetse species [14,18,19], attraction to visual targets of different colours was modelled using calculated photoreceptor responses as predictor variables. In these studies, tsetse attraction could be explained by a mechanism to which the R7y photoreceptor contributes positively, and the R8y and R7p receptors contribute negatively (see Fig 1A) [21]. Such a mechanism explains the greater attractiveness of blue and black fabrics versus alternatives, and the marked unattractiveness of UV reflecting fabrics [8,14,18,19]. These receptor-based models, therefore, permit a colorimetric approach to fabric colour engineering for improved attractiveness to tsetse, but to facilitate that aim some further exploration is required.

Tsetse photoreceptor sensitivities are indeed broadly similar to those of *Musca* and *Calliphora*, as was assumed in earlier work [29]. However, differences in detail deserve consideration and may have ramifications for photoreceptor-based behavioural explanations. Electrophysiological work on laboratory-reared *G*. *m*. *morsitans* found that the R1-6 photoreceptor class had its ‘green’ sensitivity peak at approximately 10 nm longer than the equivalent receptor class of *Musca* [29] (Fig 1B). In addition, the R7y and R8y photoreceptors of *G*. *m*. *morsitans* had broader sensitivity functions with greater sensitivity to blue wavelengths than those of *Musca*, due to a complete lack of diet-derived C_40_ carotenoid screening pigment in the R7y rhabdoms [29] (Fig 1C). Biochemical analysis of retinae from *G*. *palpalis palpalis* reared on a different diet did recover C_40_ carotenoids at about one third the concentration normally found in *Calliphora*, which would likely result in somewhat screened photoreceptor responses closer to the generic sensitivity functions, but no accompanying electrophysiological work was conducted [29]. Therefore, the differing R7y and R8y sensitivities of *G*. *m*. *morsitans* appear to have resulted from diet. However, due to the unique life history of tsetse in which nutrition is only via animal blood and larvae do not feed, it is certainly plausible that spectral sensitivities vary between the extremes of screened and unscreened sensitivities in wild populations, according to the vertebrate hosts locally available. In addition, earlier work assumed that all photoreceptor responses could contribute to behaviour through a single mechanism that summed their weighted excitations (c.f. [32]), partly because recent work on *Drosophila* has demonstrated that all receptor classes can contribute to colour discrimination in that species [33]. However, the R1-6 and R7-8 photoreceptors are anatomically distinct and have long been hypothesised to supply segregated, parallel processing channels in which the R7-8 photoreceptors provide chromatic information contributing to colour vision, and the R1-6 photoreceptors provide achromatic (luminance) information [27,34,35,36]. Furthermore, conditioned colour discrimination experiments with the blowfly *Lucilia* sp. suggest comparison of R7p versus R8p, and R7y versus R8y responses in separate opponent channels for the two types of ommatidia, with categorical encoding of just four discriminable colours based upon the signs of each of these photoreceptor response comparisons [37]. Although this categorical model of colour perception is yet to be shown experimentally for any fly other than *Lucilia*, it has become the most widely applied model of fly colour vision (see [27], and references therein), and should also be considered for tsetse.

### The aims of this study

The principal aim of the work reported in this paper was to develop photoreceptor-based models of tsetse attraction by exploring modified assumptions about the visual sensitivities of tsetse and the neurophysiological organisation of their visual system. This was done using an existing set of field data on the attraction of *G*. *f*. *fuscipes* to fabric panels of a range of different colours, since this species is considered the most important vector of Gambian HAT and the study provided the only detailed investigation of tsetse attraction to tiny targets of different colours [14]. The goal was not to determine which assumptions about the tsetse visual system were closest to the true situation in the population under investigation, but to find out whether robust colorimetric principles for fabric optimisation could be determined regardless of these assumptions. The work has direct application in the engineering of polyester fabrics for optimal tsetse attraction, and to facilitate this, calculation tools are provided with this paper and the ways in which they can be employed in fabric colour engineering are explained.

## Methods

### Source data

Analyses were performed on a large dataset reporting the attraction of *G*. *f*. *fuscipes* to a total of 37 tiny targets of different colours in 15 separate experiments conducted on Chamaunga Island, Lake Victoria, Kenya [14]. Unlike the tiny targets employed in control operations where fabric and net panels are impregnated with insecticide, tsetse were sampled in this experimental study using grids of electrocuting wires overlaying the fabric and net portions of the target [14]. The fabrics tested in these experiments included phthalogen blue and black cotton fabrics, and a variety of polyesters including examples of ‘phthalogen blue’ and royal blue polyesters similar to those used in the production of tsetse traps and targets [14]. This dataset was one of three analysed in initial attempts to develop a receptor-based model of tsetse attraction [21,22].

Each experiment compared tsetse catches at a sample of five tiny targets of different colours, one of which was always a phthalogen blue cotton standard [14]. The experimental design comprised two Latin squares of five days by five sites, and the total number of male and female tsetse caught at each target over the duration of the experiment was directly reported (‘sample sizes’ in the right hand columns of tables 1 and 2 in the source publication) [14]. This contrasts with the reporting of ‘catch indices’ alone (the de-transformed mean catch of each target expressed as a proportion of that at the phthalogen blue cotton standard), which were used in initial receptor-based modelling work [21].

The reflectance spectrum for each fabric in the original study was provided by the authors as online supplementary materials [14]. These spectra quantified fabric reflectance from 190 nm to 900 nm, at 10 nm increments.

### Calculating photoreceptor excitations

Photoreceptor excitations can be computed from the reflectance spectra of the stimuli of interest and a number of additional input functions [21,24,25]. Reflectance spectra for each tiny target, *I*_*t*_*(λ)*, were linearly interpolated to achieve 2 nm resolution, and transformed to express reflectance as a proportion. Green leaves were assumed to provide the background to these targets, and this assumption is justified by photographs of similar tsetse-sampling equipment set up for field trials [12,13]. The typical green leaf function of [25], linearly interpolated for 2 nm resolution, was thus employed as the background reflectance spectrum, *I*_*b*_*(λ)*. As a daylight illuminant function, *D(λ)*, the CIE standard D65 was employed, linearly interpolated for 2 nm resolution, converted to photon units as in [24], and normalised to a maximum of unity. In this analysis, all functions were rounded to three decimal places. A spreadsheet that includes these functions and conducts the calculations described is provided (S1 File).

In addition to the above, spectral sensitivity functions, *S(λ)*, are required for each photoreceptor class, and an important aim of this study was to investigate two extreme assumptions about tsetse spectral sensitivities. In earlier work, well-established sensitivity functions for *Musca* and *Calliphora* were employed for this purpose, extracted from [26] using Data Thief software [38] (Fig 1A). For the current work, these functions were extrapolated beyond the original 310–600 nm range. In addition, measured R1-6, R7y, and R8y spectral sensitivities for *G*. *m*. *morsitans* were extracted from [29] using Data Thief, and the available data points connected by linear interpolation, before extrapolation and combination with data from the generic functions for *Musca* and *Calliphora*. The full sensitivity spectra are illustrated in Fig 1B and 1C, and further details on their construction are provided in S1 Text.

Based on the above input functions, the effective photon catch (*P*) of reflected light from a given tiny target, was calculated for each of a tsetse’s five photoreceptor classes (*r*), as follows:

$$P_{r}=R_{r}\int_{300}^{700}I_{t}(\lambda )S_{r}(\lambda )D(\lambda )d\lambda$$

*R* is a range sensitivity factor that adjusts the sensitivity of each photoreceptor such that background stimulation would elicit a half maximal response in each photoreceptor class, representing photoreceptor adaptation:

$$R_{r}=1/\int_{300}^{700}I_{b}(\lambda )S_{r}(\lambda )D(\lambda )d\lambda$$

The resulting photon catches were non-linearised to represent the transduction process in each photoreceptor, providing excitation (*E*) based upon:

$$E_{r}=P_{r}^{n}/(P_{r}^{n}+1)$$

The exponent, *n*, was set to 1.0, as occurs in fully light adapted photoreceptors [39] (c.f. [25,40]).

### Indices representing opponent processing

Photoreceptor excitations calculated as above were used in some models of tsetse attraction. In addition, and in order to compare structured models of photoreceptor excitation processing, a number of additional predictor variables were computed from calculated photoreceptor excitation values. These predictor variables were chosen to represent specific hypotheses about the organisation of the fly visual system, and not to evaluate all possible organisations of opponent processing and determine the most likely (c.f. [40]).

To represent the potential organisation of photoreceptor excitations into separate opponent mechanisms for the R7 and R8 photoreceptors of ‘y’ and ‘p’ type ommatidia [27,37], a range of indices were calculated and evaluated (described in S2 Text). The index that provided the best fit to the data and is presented in the main text was calculated as follows:

$$Opp_{s}=E_{R7_{s}}/E_{R8_{s}}$$

Where *s* denotes the ‘y’ or ‘p’ opponent system.

In order to represent the categorical encoding of these separate opponent mechanisms [37], the above index was re-coded as 0 if <1.0, and 1 otherwise. The evaluation of models including these categorical predictor variables is fully described in S2 Text.

Finally, the calculated excitations of the R7-8 photoreceptors were each expressed relative to the summed excitation across all four such receptors, in order to represent a generic encoding of colour information separated from luminance:

$$Rel_{r}=E_{r}/(E_{R7p}+E_{R7y}+E_{R8p}+E_{R8y})$$

Where *r* here denotes photoreceptors R7p, R7y, R8p, or R8y only.

### Statistical analyses

The availability of actual tsetse catches, rather than catch indices alone, permitted a refined statistical analysis versus previous work [21]. In overview, the aim was to explain tsetse catches in the complete dataset, based upon the photoreceptor excitations that would be elicited when a tsetse viewed each tiny target. Analyses were conducted in four separate blocks comprising separate analyses of male and female tsetse catches, and within these, separate analyses using photoreceptor excitations calculated using screened (Fig 1B) and unscreened (Fig 1C) sensitivity functions.

The original dataset comprised 15 separate experiments and it was logical to assume that tsetse catches at different tiny targets within the same experiment would be related. This might happen if, for example, the number of tsetse in the local area varied between experiments. For this reason, Generalised Estimating Equations were employed to account for these experimental clusters within the complete dataset [41,42,43]. These analyses were implemented using the GENLIN procedure of IBM SPSS version 22.0.0.2 (IBM Corp., Armonk NY, USA).

I chose the total number of tsetse sampled at each tiny target over the course of an experiment as the response variable, and since this variable is a count, it was assumed to follow a negative binomial distribution. The negative binomial distribution provides an alternative to the Poisson distribution for count data where the variance cannot be assumed to equal the mean [43]. The negative binomial distribution is specified by a dispersion parameter, *k*, which was estimated for the saturated five photoreceptor predictor model in each analysis block via the GENLIN procedure, and then fixed at that value for investigation of all models within that analysis block (i.e. all models explaining the same response variable based upon subsets of the same predictors, or indices calculated from them; see S2 Text).

Since each experiment used two Latin squares of five days by five sites for each tiny target colour, the total tsetse catch of each tiny target was assumed to be equally related to that of every other within the same experiment (i.e. effects of relative target position and local fly depletion within each experimental cluster were controlled for by its design). As such, the correlation matrix representing this dependence within each experimental cluster was specified as exchangeable. A log link function was specified, similar to earlier analyses [14,19,21].

An information-theoretic approach to model evaluation and selection was employed [44]. This was based upon the corrected quasi-likelihood under independence model criterion, QICC, which is a modification of AIC (Akaike’s Information Criterion) for use with GEE that is corrected for small sample sizes with a stricter penalty for model complexity [43,45,46]. In order to simplify the main text of this manuscript, model selection methods and results are fully described in S2 Text, whilst the best-fitting models from each stage of analysis are described in the main text.

## Results

### The effect of modified spectral sensitivity functions on calculated photoreceptor excitations

For the 37 fabric reflectance spectra under investigation, photoreceptor excitation values calculated with the tsetse-like R1-6 sensitivity function (Fig 1B and 1C) were strongly correlated with those calculated using the generic R1-6 sensitivity function (Fig 1A), (Spearman’s rank correlation; r_s_ = 0.992, p<0.001, N = 37). Similarly, photoreceptor excitation values calculated using the unscreened, *G*. *m*. *morsitans* R7y sensitivity function (Fig 1C) were strongly correlated with those calculated using the screened, generic R7y sensitivity function (Fig 1B), (r_s_ = 0.988, p<0.001, N = 37); and the same was true of unscreened and screened R8y sensitivity functions (Fig 1C and 1B respectively; r_s_ = 0.951, p<0.001, N = 37). As such, modified spectral sensitivity assumptions resulted in only subtle differences in the excitation values calculated for individual photoreceptor types.

Generic sensitivity functions for the R7 and R8 photoreceptors (with carotenoid screening of R7y and R8y), and the R1-6 sensitivity function based upon data from *G*. *m*. *morsitans*, were used to represent the hypothesised spectral sensitivities of a non-carotenoid-deprived tsetse (henceforth ‘screened sensitivities’; Fig 1B). Substitution of the unscreened R7y and R8y sensitivity functions recorded from *G*. *m*. *morsitans* into this set represented the known spectral sensitivities of a carotenoid-deprived tsetse (henceforth ‘unscreened sensitivities’; Fig 1C). Within each complement of assumed sensitivities, particularly strong correlations (r_s_>0.9) were present between R1-6 excitations and both R8p and R8y excitations, and between R7y and R8p excitations (Tables 1 and 2). However, these correlations were generally stronger within the unscreened sensitivity set, which also included strong correlations between R1-6 and R7y excitations, and R8y and R8p excitations (Table 2).

**Table 1:** Spearman’s rank correlation coefficients for pairs of screened photoreceptor excitations calculated from the reflectance spectra of 37 fabrics in [14].

	E_R1-6_	E_R7p_	E_R7y_ (s)	E_R8p_	E_R8y_ (s)
E_R1-6_					
E_R7p_	0.685				
E_R7y_ (s)	0.880	0.733			
E_R8p_	0.947	0.604	0.927		
E_R8y_ (s)	0.932	0.668	0.741	0.821	

All correlations are significant at p<0.001; Spectral sensitivity functions were the screened (denoted by ‘s’, above) functions from Fig 1B.

**Table 2:** Spearman’s rank correlation coefficients for pairs of unscreened photoreceptor excitations calculated from the reflectance spectra of 37 fabrics in [14].

	E_R1-6_	E_R7p_	E_R7y_ (u)	E_R8p_	E_R8y_ (u)
E_R1-6_					
E_R7p_	0.685				
E_R7y_ (u)	0.912	0.686			
E_R8p_	0.947	0.604	0.966		
E_R8y_ (u)	0.989	0.665	0.865	0.920	

All correlations are significant at p<0.001; Spectral sensitivity functions were the unscreened (denoted by ‘u’, above) functions from Fig 1C.

### Modelling attraction based upon weighted photoreceptor excitations

I first modelled *G*. *f*. *fuscipes* attraction based upon weighted combinations of between one and five photoreceptor excitations computed using screened sensitivity functions. The best-fitting model common to the male and female datasets employed the excitations of R7y, R7p, and R8y as predictor variables (Table A in S2 Text). The coefficients that translate these photoreceptor excitations into the predicted natural log-transformed tsetse catch of a tiny target are given in Table 3. These coefficients indicated a positive influence of R7y, and negative influences of R7p and R8y, on tsetse attraction (Table 3; S1 and S2 Figs).

**Table 3:** Coefficients for models of tsetse attraction based upon weighted photoreceptor excitations.

	Intercept		E_R1-6_		E_R7p_		E_R7y_		E_R8p_		E_R8y_	
	B_0_	Wald Χ^2^_1_	B_1_	Wald Χ^2^_1_	B_2_	Wald Χ^2^_1_	B_3_	Wald Χ^2^_1_	B_4_	Wald Χ^2^_1_	B_5_	Wald Χ^2^_1_
		(p)		(p)		(p)		(p)		(p)		(p)
**(A) Males**												
*(i) Screened*	5.153	1911.831			-1.847	62.245	1.985	39.585			-1.211	22.711
		**(<0.001)**				**(<0.001)**		**(<0.001)**				**(<0.001)**
*(ii) Unscreened*	5.301	1716.452	17.330	8.019	-2.171	22.669			-3.872	3.341	-12.593	10.939
		**(<0.001)**		**(0.005)**		**(<0.001)**				(0.068)		**(0.001)**
**(B) Females**												
*(i) Screened*	5.456	2964.607			-2.283	199.677	2.031	60.839			-1.118	28.929
		**(<0.001)**				**(<0.001)**		**(<0.001)**				**(<0.001)**
*(ii) Unscreened*	5.607	5133.699	17.454	37.104	-2.593	123.653			-3.980	18.662	-12.481	44.784
		**(<0.001)**		**(<0.001)**		**(<0.001)**				**(<0.001)**		**(<0.001)**

B is the coefficient estimated for the predictor variable in its column heading. Wald Χ^2^ tests assess whether these coefficients are significantly different from zero. Coefficients combine with calculated values of the predictor variables to provide a prediction of ln(tsetse catch) for a tiny target. For example, for male tsetse and assuming screened photoreceptor sensitivities (A)(i), ln(male catch) = B_0_ + B_2_E_R7p_ + B_3_E_R7y_ + B_5_E_R8y_.

I next repeated this analysis using photoreceptor excitations computed using unscreened sensitivity functions. Although this analysis provided moderate support for the +R7y -R7p -R8y model, the best-supported model common to the male and female datasets included the excitations of R1-6, R7p, R8p, and R8y (Table B in S2 Text). Like the +R7y -R7p -R8y model implicated using screened sensitivity functions, these models included significant negative coefficients for R7p and R8y responses (Table 3). However, rather than including a positive effect of R7y excitation, these models included positive effects of R1-6 excitation and an additional negative (though not always significant) effect of R8p excitation (Table 3). Due to the similarity between unscreened R7y and R1-6 sensitivity functions at short wavelengths (Fig 1C), and the enhanced negative effect of R8p and unscreened R8y at longer wavelengths (Fig 1C; Table 3), the functional effect of this combination of predictors was qualitatively similar to that of the three predictors in the screened models (see also S1 Fig, S2 Fig).

### Evaluating alternative models of fly colour vision

I next evaluated a range of models that incorporated the structured processing of photoreceptor excitations. In contrast to the simple, weighted photoreceptor excitation models above, the most widely applied model of fly colour vision is based upon categorically encoded R7y versus R8y, and R7p versus R8p opponent mechanisms [27,37]. However, such categorical predictor variables alone provided a poor fit to the attraction data (Table C in S2 Text). When R1-6 excitations were added to these models to represent the evaluation of luminance separate to the categorical encoding of colour, their fit was improved substantially. Despite this, none of these models was competitive with the weighted photoreceptor excitation models (Table C in S2 Text).

The next set of models considered the possibility that photoreceptor excitations were processed in the previously described opponent channels, but without categorical encoding of their outputs. Calculated in a variety of different ways (S2 Text), a pair of opponent comparisons alone provided a relatively poor fit to the data that was not competitive with the weighted photoreceptor excitation models (Table D in S2 Text). However, the fit of these models was greatly improved by the addition of the R1-6 photoreceptor excitation representing a separate luminance channel (Table D in S2 Text). The fit of these models to the data was better than the weighted photoreceptor excitation models when either screened or unscreened photoreceptor sensitivities were assumed. Further, these models included the best supported of all models examined for the male data (Table D in S2 Text; S1 and S2 Figs). In all such models, R1-6 excitation had a significant, negative coefficient (Table 4; S1 Table). The various representations of the ‘p’ opponent system had a significant negative coefficient (Table 4; S1 Table), indicative of a negative effect of R7p excitation and a positive effect of R8p excitation on attraction. The effect of the ‘y’ opponent system was not always significant in the best-fitting model of this kind (Table 4), but was in models that represented this opponent interaction using different computations (S1 Table). Nevertheless, the sign of the ‘y’ opponent system coefficient was positive in all models (Table 4; S1 Table), consistent with a positive effect of R7y excitation, and a negative effect of R8y excitation, on tsetse attraction. Thus, these models shared similarities with the weighted photoreceptor excitation models in terms of the nature of the effect of individual classes of photoreceptor on tsetse attraction (Table 3)

**Table 4:** Coefficients for models of tsetse attraction based upon separate ‘y’ and ‘p’ opponent systems.

	Intercept		E_R1-6_		Opp_y_		Opp_p_	
	B_0_	Wald Χ^2^_1_	B_1_	Wald Χ^2^_1_	B_2_	Wald Χ^2^_1_	B_3_	Wald Χ^2^_1_
		(p)		(p)		(p)		(p)
**A. Males**								
*(i) Screened*	6.437	421.759	-1.698	34.847	0.169	4.722	-0.862	56.415
		**(<0.001)**		**(<0.001)**		**(0.030)**		**(<0.001)**
*(ii) Unscreened*	6.569	415.070	-1.839	41.088	0.191	2.457	-0.894	58.727
		**(<0.001)**		**(<0.001)**		(0.117)		**(<0.001)**
**B. Females**								
*(i) Screened*	7.082	1041.614	-2.135	105.575	0.098	2.143	-1.023	161.362
		**(<0.001)**		**(<0.001)**		(0.143)		**(<0.001)**
*(ii) Unscreened*	7.156	961.315	-2.213	117.439	0.110	1.004	-1.041	169.529
		**(<0.001)**		**(<0.001)**		(0.316)		**(<0.001)**

Table conventions and interpretation as for Table 3.

Finally, I analysed a model in which the relative excitations of the four R7-8 photoreceptors were used as predictor variables, intended to represent a generic encoding of colour information separated from luminance (Table D in S2 Text). Without the addition of R1-6 excitation to represent a separated luminance channel the fit to the data was poor. However, with the addition of R1-6 excitation this was among the best-fitting models for male data and was the strongest supported of all models for female data (Table D in S2 Text; S1 and S2 Figs). Interpretation of the coefficients from such analyses is complicated because relative photoreceptor excitations are proportions, and thus a change in any relative photoreceptor excitation must cause a change in at least one other. Thus, coefficients are presented for models with each relative excitation value excluded in turn. The interpretation of the coefficient for each relative photoreceptor excitation is thus the change in predicted natural log-transformed tsetse catch resulting from an increase in the relative excitation of the photoreceptor in question with a concomitant decrease in that of the omitted photoreceptor (Table 5). In all models, the R1-6 luminance channel was a significant negative predictor of attraction (c.f. Table 4). A negative influence of relative R7p excitation on attraction was strongly supported by its negative coefficient in all of its models, meaning that an increase in the relative excitation of R7p and a decrease in that of any other photoreceptor, resulted in decreased tsetse attraction; this is also supported by the positive coefficients of all relative excitations where R7p was excluded, meaning that an increase in relative excitation of any photoreceptor at the expense of R7p increases tsetse attraction (Table 5). The opposite pattern was observed for photoreceptor R7y, strongly supporting a positive influence of relative R7y excitation on tsetse attraction (Table 5). Relative R8p and R8y excitations had a less clear-cut influence on attraction, with the sign of their coefficients depending on the photoreceptor omitted (Table 5). Thus, once again, the individual contributions of the R1-6, R7y and R7p classes were similar to those suggested by the previously considered models.

**Table 5:** Coefficients for models of tsetse attraction based upon relative R7-8 excitations.

	A. Males				B. Females			
*(i) Screened*								
Omitted	Rel_R7p_	Rel_R7y_	Rel_R8p_	Rel_R8y_	Rel_R7p_	Rel_R7y_	Rel_R8p_	Rel_R8y_
Intercept	1.102	**9.551**	**6.552**	**3.948**	-1.062	**13.650**	**4.353**	**5.531**
E_R1-6_	**-1.172**	**-1.172**	**-1.172**	**-1.172**	**-1.518**	**-1.518**	**-1.518**	**-1.518**
Rel_R7p_		-8.449	**-5.451**	-2.846		**-14.711**	**-5.414**	**-6.593**
Rel_R7y_	8.449		2.998	**5.603**	**14.711**		**9.297**	**8.119**
Rel_R8p_	**5.451**	-2.998		2.605	**5.414**	**-9.297**		-1.178
Rel_R8y_	2.846	**-5.603**	-2.605		**6.593**	**-8.119**	1.178	
*(ii) Unscreened*								
Omitted	Rel_R7p_	Rel_R7y_	Rel_R8p_	Rel_R8y_	Rel_R7p_	Rel_R7y_	Rel_R8p_	Rel_R8y_
Intercept	1.037	**11.837**	4.587	**4.180**	-0.503	**16.644**	0.577	**6.097**
E_R1-6_	**-1.310**	**-1.310**	**-1.310**	**-1.310**	**-1.617**	**-1.617**	**-1.617**	**-1.617**
Rel_R7p_		**-10.800**	-3.550	-3.143		**-17.147**	-1.080	**-6.601**
Rel_R7y_	**10.800**		7.250	**7.657**	**17.147**		**16.068**	**10.547**
Rel_R8p_	3.550	-7.250		0.407	1.080	**-16.068**		-5.521
Rel_R8y_	3.143	**-7.657**	-0.407		**6.601**	**-10.547**	5.521	

Coefficients highlighted with bold text are significant at p<0.05. Interpretation of these coefficients is as for Table 3.

### General principles for the improvement of attractive polyesters

It is beyond the available data to evaluate the true physiological mechanism of tsetse attraction, and the value of the above models is in their similar predictions about the contribution of individual photoreceptor classes to attraction: the best-fitting models supported negative influences of R7p and R8y excitation on attraction, and positive influences of R7y excitation on attraction (Tables 3–5). All models in which R1-6 excitation provided a separate luminance channel suggested a negative influence on attraction (Tables 4 and 5).

**Fig 2:**
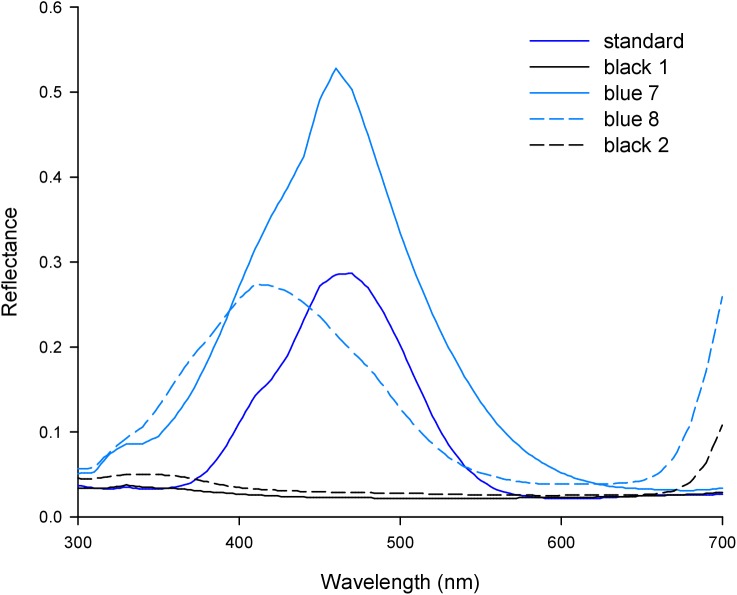
Reflectance spectra reported for five fabrics of applied importance by [14]. Fabric names are those used in the source publication. ‘Standard’ is a phthalogen blue cotton material, and ‘black 1’ a black cotton, both of which are common fabrics in previous studies of tsetse attraction. ‘Blue 7’ is a phthalogen blue-like polyester, ‘blue 8’ a royal blue polyester, and ‘black 2’ a black polyester, all representative of polyesters used in modern tsetse attractant devices. Reflectance spectra were originally reported by [14], linearly interpolated and rounded by the author (see methods).

**Fig 3:**
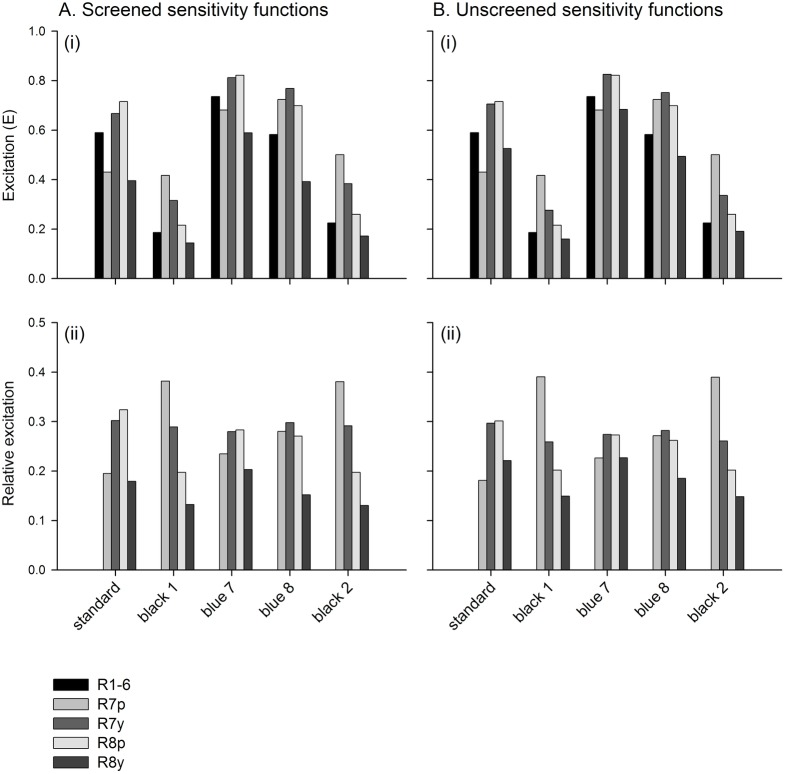
Photoreceptor responses calculated from the reflectance spectra in Fig 2. Photoreceptor excitations were calculated using the screened (A) and unscreened (B) sensitivity functions shown in Fig 1. The excitation values for all five receptor types are plotted in the upper panels (i). In the lower panels, the relative excitations of each R7-8 photoreceptor are represented as a proportion of the summed excitation across all four such photoreceptors (ii).

In order to illustrate these common principles I conducted a graphical analysis of experiment eight reported by [14] which compared the tsetse catches of five fabrics with particular applied importance. These comprised two traditional cotton materials that were available from previous studies of tsetse target design, a standard phthalogen blue (standard) and a black (black 1); and three polyester materials used in the production of modern tsetse targets, a phthalogen-like blue (blue 7), a royal blue (blue 8), and a black (black 2). The reflectance spectra for these fabrics are shown in Fig 2, and the calculated excitations of screened and unscreened fly photoreceptors to these fabrics, expressed both as excitation values for photoreceptors R1-8, and as relative excitations across the R7-8 photoreceptors, are shown in Fig 3.

**Fig 4:**
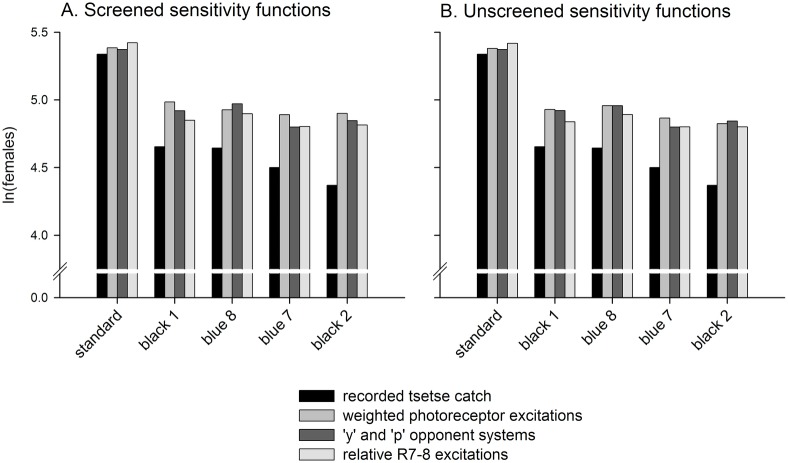
Actual and predicted tsetse catches from a single experiment of [14]. In each panel, natural log-transformed tsetse catches are plotted from experiment eight of [14], and the stimuli have been ordered by this measurement. Alongside these are predicted natural log-transformed tsetse catches computed using screened (A) and unscreened (B) photoreceptor excitation values, and coefficients from the best-fitting receptor-based models of attraction. The weighted photoreceptor excitation model differs between panels in the number and identity of its predictor variables, and these models are those reported in Table 3. The ‘y’ and ‘p’ opponent system models are those reported in Table 4. The relative R7-8 excitation models are those reported in Table 5. In this experiment, tsetse catches were lower than predicted using the population-averaged coefficients derived from GEE analysis, an effect of experimental clustering (see also S1 Fig and S2 Fig).

Fig 4 shows the natural log-transformed catches of female *G*. *f*. *fuscipes* (only) from this single experiment, and the different tiny targets are now re-ordered according to their relative attractiveness. The coefficients from the best-fitting GEE models (Tables 3–5), have been used to compute predicted log-transformed catch values for each tiny target based upon the photoreceptor excitation values in Fig 3. In this experiment, less tsetse were caught than the models predicted (see also S1 Fig and S2 Fig for an illustration of such experimental clustering across the complete dataset), but the pattern of target attractiveness was effectively predicted by all candidate models. A qualitative analysis of the factors that bring about this pattern provides a means to illustrate the similarity of the models generated in this analysis, and general principles for the future optimisation of fabrics for tsetse attraction.

Firstly, the best-supported models (excluding the weighted photoreceptor excitation model using unscreened spectral sensitivities; Tables 3–5), generally indicate that fabrics are most attractive to tsetse when they maximise relative excitation of the R7y and possibly R8p photoreceptors, and minimise that of the R7p and R8y photoreceptors. In practical terms this means reflection of light between ca. 380 nm and ca. 500 nm, and minimal reflection outside of this range (ignoring reflection at >650nm, to which flies are not sensitive), (Fig 1). This pattern of reflectance and photoreceptor excitation is strongly evident for the phthalogen blue cotton standard fabric in Figs 2 and 3, and versus this pattern it can clearly be seen that the black fabrics elicit relatively higher excitation in the R7p photoreceptor than in R7y or the R8 photoreceptors, providing an explanation for their lesser attractiveness (Figs 3 and 4). Of the blue fabrics, blue 8 differs from the others in that it reflects maximally at a shorter wavelength, resulting in greater reflectivity of UV wavelengths (Fig 2), and higher relative excitation of R7p (Fig 3). This might explain its reduced attractiveness to tsetse versus the phthalogen blue standard (Fig 4). Blue 7, meanwhile, appears to provide a relatively attractive ratio of excitation in R7y and R8p, versus that in R7p and R8y, although this pattern is not as marked as for phthalogen blue cotton (Fig 3).

A second factor determining attractiveness implicated by the models presented is that of overall brightness. In the best models using weighted photoreceptor excitations this comes about because negative coefficients sum to a greater absolute value than positive ones in each model (Table 3). Therefore, if all photoreceptors responded equally to a given stimulus, predicted natural log-transformed tsetse catch would have a negative relationship with the strength of their excitation (i.e. negative effects dominate, and reducing them even at the expense of positive effects makes the target more attractive). In the models where colour is represented separately to luminance (Tables 4 and 5), the broadband R1-6 photoreceptor’s excitation is always a negative predictor of attraction. This principle can explain the lesser attractiveness of blue 7 versus the phthalogen blue standard because this fabric elicits strong responses in all photoreceptors including R1-6 (Figs 3A and 4). The same principle in reverse explains the relatively higher attractiveness of the black fabrics than might be expected from the pattern of excitation across the R7 and R8 photoreceptors alone (Fig 3). The rising reflectance functions of blue 8 and black 2 above 650nm are immaterial, because flies have no sensitivity to light in this region of the spectrum (Figs 1 and 2).

Thus, regardless of whether screened or unscreened sensitivity functions are assumed, or which photoreceptor-based model of attraction is chosen, the recommendations for fabric optimisation are that: (i) reflectance should be minimised in the regions that strongly excite R7p and R8y (< ca. 380 nm, and > ca. 500 nm) relative to the regions that most strongly excite R7y and R8p (ca. 380 nm < > ca. 500 nm); and (ii) the overall reflectance of the fabric should be relatively low.

## Discussion

This paper presents photoreceptor-based models of tsetse attraction to tiny targets of different colours under differing assumptions about the spectral sensitivities of tsetse photoreceptors and the organisation of the fly visual system. Individual photoreceptor excitation values calculated using spectral sensitivities based on those recorded for *G*. *m*. *morsitans* lacking in carotenoid screening pigments were strongly correlated with those calculated for the same type of photoreceptor using the screened spectral sensitivities of *Musca* and *Calliphora*, as in earlier work [21]. However, correlations between the excitation values of different types of photoreceptor were stronger when the unscreened spectral sensitivities of *G*. *m*. *morsitans* were assumed, and the best models based on weighted photoreceptor excitations differed under the assumptions of screened and unscreened spectral sensitivity. However, regardless of these assumptions, photoreceptor-based models of attraction that separated chromatic (R7-8) from achromatic (R1-6) channels fitted the data better than those models that combined weighted photoreceptor excitations within a single mechanism, and were qualitatively similar under differing assumptions about photoreceptor spectral sensitivities. It is suggested that behaviourally relevant colorimetric values for the engineering of attractive polyester fabrics can be derived from these models, and that exploiting them to develop a polyester with the same attractiveness as phthalogen blue cotton has the potential to as much as double the efficiency of tiny targets (e.g. [14]).

### On the photoreceptor basis of tsetse attraction

Identifying the true photoreceptor basis of tsetse attraction within the *G*. *f*. *fuscipes* population investigated in the original field study was an aim beyond the data available. This is partly because the stimuli investigated in that experiment were chosen to prospect for attractive fabrics and not to evenly sample all regions of fly colour space in order to determine the mechanism of visual attraction. Thus, unravelling the photoreceptor basis of tsetse attraction will require dedicated experiments, but the models presented in this work are expected to capture important principles of these visual mechanisms useful in the applied context. Many of these principles were evident across models with different underlying assumptions.

Firstly, the best-fitting models from each stage of analysis were chromatic, in that they incorporated excitation values for more than one photoreceptor class and processed them in an opponent fashion [24,32]. These models always explained the data better than simple achromatic models using excitation values for any single photoreceptor class.

Initially, models were considered in which weighted photoreceptor excitations were combined via a hypothesised single opponent mechanism (c.f. [21,32]). Within this group of models, that preferred under screened and unscreened spectral sensitivity assumptions differed. Under screened assumptions, the preferred model was characterised by a positive influence of R7y on attraction, and negative influences of R7p and R8y, as in an earlier analysis conducted using different methods and incorporating field data for riverine and savannah tsetse [21]. However, the model preferred using unscreened sensitivity functions substituted the positive R7y effect for a positive contribution of R1-6 excitation, and an additional negative contribution of R8p excitation. This scheme was contrary to the other well-supported models in this analysis, and to intuition from the widely acknowledged principle that tsetse are attracted towards darker or bluer stimuli [8,47]. Biochemical analyses of the retinae of *G*. *f*. *fuscipes* from the original study location would be required to determine the presence or absence of screening pigments in their eyes, and thus their true spectral sensitivities [29,31]. However, because structured models were better-supported explanations for tsetse behaviour, and these were similar regardless of spectral sensitivity assumptions, it is suggested that this issue is of limited immediate importance in the applied context.

There was very little evidence to support the involvement of a categorical, four colour system in innate colour preference in tsetse, as was proposed to explain conditioned colour discrimination in *Lucilia* [37]. The same conclusion was reached in earlier work [21], but was extended here by examining different underlying spectral sensitivities, and the separate involvement of the R1-6 photoreceptors as an achromatic channel. This result might be explained if the visual mechanisms employed differed between fly species and/or behavioural contexts (innate attraction versus learned discrimination). However, the categorical fly colour model has become the most accepted model of fly colour vision and is very widely applied across species and contexts (e.g. [27], and references therein). Since this model differs to the standard models of colour vision applied by default in other species [24,48,49], it is recommended that researchers investigating the visually guided behaviour of other flies consider both categorical and alternative colour visual models, at least until a colour model is firmly established for the fly species and context under investigation.

Regardless of the photoreceptor spectral sensitivities assumed, those models that separated chromatic (comparisons of R7-8 photoreceptor excitations) from achromatic cues (actual R1-6 photoreceptor excitations) provided a better fit to the data than those models that combined weighted photoreceptor excitations with no particular assumption about their role. Furthermore, these models were qualitatively similar regardless of the spectral sensitivity functions assumed. The hypothesised organisation of the fly visual system into segregated chromatic and achromatic systems has been proposed for some time based upon anatomical and physiological evidence [34,35,36]. However, there is also increasing anatomical, physiological, and behavioural evidence for the interaction of these pathways and against complete segregation [33,34,50]. It should also be noted that the current analyses focussed on particular hypotheses about opponent coding and did not seek to investigate the plausibility of all possible organisations (c.f. [40]). Thus, the models presented in this analysis provide physiologically plausible, but likely simplistic, explanations for tsetse behaviour suited to use in fabric colour engineering.

An important additional consideration is that each of the best-fitting models of attraction described in this paper incorporates inputs from R7 or R8 receptors of ‘y’ and ‘p’ types (although the ‘y’ opponent system effect was not always significant in the best ‘y’ and ‘p’ opponent system models). These ‘y’ and ‘p’ type receptors are located in separate ommatidia and are randomly distributed over the eye [26]. Considering partial interommatidial angles in the horizontal plane ranging from 0.6 to 1.7° (across males of *Calliphora* and *Musca* combined [28]), and angular sensitivity functions with widths at 50% sensitivity of 1.7° for R7y and 2.7° for R1-6 (for *G*. *m*. *morsitans*, [29]), a target would presumably need to subtend greater than ca. 4° at the eye in order to effectively excite photoreceptors of neighbouring ommatidia. Given the size of tiny targets, this could occur at a maximum range of ca. 3.6 m. Because chromatic cues also have limited range for foraging honeybees, they utilise green photoreceptor contrast to guide their initial approach towards flowers [51], so it is possible that the initial approach of tsetse towards targets is also elicited by simpler visual cues. However, after approaching a target tsetse characteristically circle in its vicinity [52], and often depart without landing on it [8,53], so their natural behaviour is certainly consistent with the evaluation of targets at relatively close range.

Of greatest relevance to the applied context of tsetse control, a set of common features were evident across all models in the analysis (though not necessarily all evident in each individual model). These principles were that tsetse were attracted to targets that maximised excitation of the R7y and possibly R8p photoreceptors relative to the R7p and R8y photoreceptors, and when the overall brightness of the fabric was relatively low. These mechanistic principles explain the widely-acknowledged attraction of tsetse towards blue and black targets, and the ineffectiveness of other target colours, particularly those with high UV reflectance [8,18,19,21]. As such, calculated excitations for fly photoreceptors, and combinations of them in the best-supported photoreceptor-based models can provide behaviourally relevant colorimetric values for the engineering of more-attractive polyester fabrics for use in tiny targets.

### Procedures for fabric colour optimisation

Before describing the ways in which receptor-based models can facilitate fabric colour optimisation, it should first be emphasised that a highly attractive colour is not the only important property of an optimised fabric. Although not the subject of this analysis, specular reflectance (mirror-like shininess) appears to decrease tsetse attraction, and can result from the fine weave of some synthetic fabrics [20]. As such, the weave and texturisation of the fabric are important considerations. Furthermore, attractive combinations of fabric and dyes must also display high levels of colour fastness under field conditions, as phthalogen blue cotton does [8]. Nevertheless, because phthalogen blue cottons attract tsetse often twice as effectively as some blue polyesters [14], colour engineering of polyesters for greater attractiveness holds considerable potential for tiny target improvement.

In the absence of receptor-based models, attempts could be made to engineer polyester fabrics with reflectance spectra matched to that of phthalogen blue cotton. This can be tackled using single-constant Kubelka-Munk theory which relates the spectral reflectance of a dyed fabric sample at a particular wavelength to K/S, the ratio of absorbance, K, to scattering, S, coefficients [23]. Because K/S values scale approximately linearly with dye concentration, the relationship between K/S and dye concentration at each wavelength can be determined experimentally. Dye recipes can then be modelled on the assumption that the K/S values of the different dyes in the mixture at any particular wavelength sum, resulting in a theoretical K/S spectrum that can be related to its probable reflectance [23]. Such an approach might determine a dye mixture that produces a reflectance spectrum matched to that of phthalogen blue cotton. However, this approach fails when the spectrum of interest cannot be matched exactly. This can occur if the same dyes are not available, as is the case for the phthalogen blue dye which can only be applied to cotton and not polyester [14]. Because colour perception depends on photoreceptor responses and not directly on reflectance spectra, large mismatches in some regions of a reflectance spectrum may be unimportant, whilst small mismatches elsewhere may result in large differences in appearance to the intended viewer [23]. The tsetse-specific receptor-based models described in this paper provide a solution to this problem.

Metamerism is the phenomenon by which spectrally dissimilar stimuli can evoke the same visual response, by virtue of eliciting a similar pattern of excitation in the viewer’s photoreceptors [23]. Thus, rather than trying to match the reflectance spectrum of interest, colorimetric colour matching approaches attempt to match values that represent the responses of the viewer’s photoreceptors [23]. Tristimulus values calculated using CIE standard observer functions represent human cone photoreceptor responses, and allow colour matching to the human eye [23]. Unfortunately, these values are irrelevant to fly vision due to the differing spectral sensitivities of their photoreceptors. However, fly photoreceptor excitation values can be calculated using the tools provided in the supporting information of this paper (and easily adjusted for different assumptions about background and illuminant), and these can provide a set of basic colorimetric values that can be employed for fly’s eye view colour matching. The findings of this work imply that it makes little difference whether screened or unscreened sensitivity functions are assumed, but all could be considered in a detailed colorimetric approach. Thus, using standard approaches modified with fly photoreceptor excitation values, it may be possible to engineer a polyester metamer of phthalogen blue cotton.

A potentially more powerful way in which the findings of this study can be applied is in polyester colour optimisation, rather than matching. The presented photoreceptor-based models provide common recommendations for the direction of change required in the calculated excitation values of each photoreceptor for increased tsetse attraction: namely, increased excitation in R7y and possibly R8p relative to R7p and R8y; and decreased excitation in R1-6. The coefficients for these models allow photoreceptor excitation values to be combined into a single value that scales with tsetse attraction. As such, once the relationship between concentration and K/S was understood for a series of candidate dyes, single-constant Kubelka-Munk theory would allow the theoretical reflectance spectra of different dye recipes to be calculated [23], from which fly photoreceptor excitations and then predicted attractiveness could be calculated. Dye recipes that maximised predicted attractiveness could then be identified, and a wide variety of computational approaches are now available to tackle such tasks. A potential advantage of this approach over colour matching is that a fabric even more effective in attracting tsetse than phthalogen blue cotton might be identified. A second advantage is that if it proved impossible to spectrophotometrically or colorimetrically match phthalogen blue cotton, an optimisation approach would yield the most attractive fabric that could be created from the candidate dyes, rather than the closest visual match. These are not necessarily the same thing because the different photoreceptor types make different contributions to attraction.

In addition to colorimetric tools for the engineering of more-attractive polyester fabrics, this paper also presented a graphical analysis of tsetse attraction and photoreceptor responses elicited by a range of specific fabrics already used in tsetse control devices [14]. This analysis suggests that a phthalogen blue-like polyester used for this purpose elicits a reasonably attractive pattern of relative responses in photoreceptors R7-8. However, the attractiveness of this fabric would be improved by decreasing its overall reflectance. This might be achieved by applying its dye in higher concentration, although the effectiveness of this will depend on the properties of the dye and base fabric. The royal blue polyester examined had high reflectance of UV wavelengths, which explained its lesser attractiveness versus phthalogen blue cotton. This fabric might be improved by addition of an optical brightener, a fluorescent dye that absorbs light at UV wavelengths and emits it in the blue, as has been suggested previously [18]. Whilst such modifications are likely to be more effective if carried out rationally and quantitatively using the previously discussed methods and the provided calculation tools, these suggestions can facilitate relatively simple and direct improvement of existing fabrics.

## Supporting information

S1 File Photoreceptor excitation calculation tool. A spreadsheet that includes the input functions described in the methods of this paper, and performs the calculations of photoreceptor excitation values described. This paper proposes that these photoreceptor excitation values, or predictions of attraction calculated from them using the coefficients in Tables 3–5, can be used as colorimetric values for fabric engineering.
(XLSX)

S1 Text Supplementary methods. A brief description of steps taken to extrapolate *Musca* sensitivity functions, and construct tsetse sensitivity functions.
(DOCX)

S2 Text Supplementary results. A full description of model selection procedures and results.
(DOCX)

S1 Fig Predicted versus actual male tsetse catches for the best-fitting GEE models. In each plot, natural log-transformed male tsetse catches from a previously published field study [14] are plotted against predicted catches computed using the coefficients generated via GEE analysis, and photoreceptor excitation values calculated from the reflectance spectra of the tiny targets used in the field study. Photoreceptor excitations were calculated using either screened (A), or unscreened (B), sensitivity functions. The models plotted are the best-fitting weighted photoreceptor excitation models (i; Table 3), ‘y’ and ‘p’ opponent system models (ii; Table 4), and relative R7-8 excitation models (iii; Table 5). Data were from 15 separate experiments within the source study, and individual data points from the same experiment have been plotted with the same symbol and colour, and are connected by straight lines. A one-to-one relationship between predicted and actual catches is indicated by the solid, straight line. Because GEE predicts the population-averaged coefficients across experiments, clustering of the data within experiments is evident in the consistently lower or higher catches within that cluster versus predicted values.
(TIF)

S2 Fig Predicted versus actual female tsetse catches for the best-fitting GEE models. In each plot, natural log-transformed female tsetse catches from a previously published field study [14] are plotted against predicted catches computed using the coefficients generated via GEE analysis, and photoreceptor excitation values calculated from the reflectance spectra of the tiny targets used in the field study. Photoreceptor excitations were calculated using either screened (A), or unscreened (B), sensitivity functions. The models plotted are the best-fitting weighted photoreceptor excitation models (i; Table 3), ‘y’ and ‘p’ opponent system models (ii; Table 4), and relative R7-8 excitation models (iii; Table 5). Data were from 15 separate experiments within the source study, and individual data points from the same experiment have been plotted with the same symbol and colour, and are connected by straight lines. A one-to-one relationship between predicted and actual catches is indicated by the solid, straight line. Because GEE predicts the population-averaged coefficients across experiments, clustering of the data within experiments is evident in the consistently lower or higher catches within that cluster versus predicted values.
(TIF)

S1 Table Coefficients for models of tsetse attraction using alternative indices to represent ‘y’ and ‘p’ opponent systems. (DOCX)

S1 Dataset Collated data used in the presented analyses. (XLSX)
