# Supplementary material for: Developing photoreceptor-based models of visual attraction in riverine tsetse, for use in the engineering of more-attractive polyester fabrics for control devices
Source: PLoS Negl Trop Dis. 2017 Mar 17;11(3):e0005448. doi: 10.1371/journal.pntd.0005448 (PMC5371378; doi:10.1371/journal.pntd.0005448)
Supplement: S1 Text — A brief description of steps taken to extrapolate Musca sensitivity functions, and construct tsetse sensitivity functions. (DOCX) [file pntd.0005448.s002.docx]

**S1 Text. Supplementary methods: Construction of photoreceptor sensitivity functions**

Initial photoreceptor-based modelling of tsetse attraction [1,2], was conducted using typical fly photoreceptor spectral sensitivity functions established for *Musca* and *Calliphora* and available between 310 and 600 nm [3,4] (henceforth, generic fly sensitivity functions). In the current work, these functions were extrapolated to allow analysis over the 300 to 700 nm wavelength range, and a new set of sensitivity functions were constructed to represent the spectral sensitivities recorded electrophysiologically for *Glossina morsitans morsitans* [5]. All sensitivity functions are presented in Fig 1 of the main paper, but this document outlines the rationale behind their construction.

**1. Short wavelength extrapolation of generic spectral sensitivity functions**

Versus the generic photoreceptor sensitivity functions used in [1], those for R1-6, R7p, R7y, and R8y were linearly extrapolated at their short wavelength end using the mean slope over a 6 nm section of the available data. In addition, that for R8p was smoothed between 400-450 nm and 470-560 nm by averaging across a data point and its two neighbours, although the peak and tails of the sensitivity function were not manipulated.

**2. Long wavelength extrapolation of generic spectral sensitivity functions, and construction of tsetse functions**

Generic sensitivity functions for R1-6 and R8y required extrapolation at the long wavelength end, and this was guided by spectral sensitivity calculations [6,7,8,9]. In addition, equivalent functions were developed for tsetse R1-6, R7y, and R8y using published data [5], combination with the generic functions, and similar spectral sensitivity calculations. The generic sensitivity functions for R7p and R8p were used to represent those of tsetse since R7p spectral sensitivity measurements are well matched between these species [5], and because no electrophysiological data are available for tsetse R8p.

**2.1 Extraction of data on tsetse spectral sensitivity**

Tsetse spectral sensitivity data were extracted from figure 6 of [5]. Data were available as 12 – 15 discrete sensitivity measurements, with each presented as means across six *G. m. morsitans* that had been raised on porcine blood (lacking in carotenoids), and three *G. m. morsitans* that had been repeatedly fed on rabbit blood [5]. Each data point was measured and extracted from the published figure using Data Thief software [10]. Since feeding on rabbit blood had little effect on photoreceptor sensitivity [3], a mean value across the two measurements was calculated at each available wavelength, weighted according to sample size. Measurements were rounded to two decimal places and linearly interpolated to achieve 1 nm resolution between the first and last data points for each photoreceptor. These functions were then sub-sampled at 2 nm resolution, and normalised to a maximum of unity.

**2.2 Generic and tsetse-specific R1-6 sensitivity functions**

Extrapolation of the R1-6 sensitivity functions at long wavelengths was guided by theoretical calculations of spectral sensitivity, *S(λ)*, to estimate the tails of the sensitivity functions. In each case, a nomogram was used to calculate alpha band absorbance of the main visual pigment (λmax 490 nm for the generic R1-6 function) [9], and adjusted for self-screening and waveguide effects [6,7,8]:

(1) $S_{i}(\lambda)=1-\exp^{-\alpha(\lambda)\cdot c\cdot l\cdot\eta(\lambda)}$

Where *α(λ)* is the calculated alpha band absorbance, *c* is photopigment concentration, and *l* is rhabdomere length. A value of *c* = 0.0065 was used, based on reported peak absorption coefficients of 0.004 - 0.009 μm^-1^ [8]. A rhabdomere length of 200 μm was used for R1-6 [6]. *η* was approximated from the waveguide parameter, *V*, by *η(V)* = 0.96 – 2.82 * exp(-1.27 * *V*) [11]. *V* was calculated by [6]:

(2) $V=(\frac{\pi\cdot d}{\lambda})\cdot(n_{r}^{2}-n_{s}^{2})^{\frac{1}{2}}$

Values of n_r_ = 1.363 and n_s_ = 1.339 were used, following [12]. Because the R1-6 rhabdomere is tapered, values of *d* distal = 2 μm, and *d* proximal = 1 μm were used, and *V* computed for both *d*s at each *λ*, and a mean *V* calculated and used to compute *η(λ)*. This function was scaled to match the amplitude of the green peak from the published generic sensitivity function. The computed values were then used to complete the long wavelength tail of the published sensitivity function from 600 to 700 nm, with no interpolation to join the two.

To construct the tsetse R1-6 sensitivity function, the generic function was duplicated up to 376 nm, replicating the fine structure of this curve in the UV resulting from the presence of a sensitising pigment and identified using recordings from *Musca* with finer wavelength resolution than those from tsetse [3,4]. At longer wavelengths, the tsetse function built from data in [5] was used, with a linear extrapolation of the tsetse curve to join that for *Musca* and avoid an adjoining decrease in the composite sensitivity function. To extrapolate the tsetse function at longer wavelengths, the procedure used for the generic function was employed with the exception that a λmax 500 nm alpha band nomogram was used [9]. This calculated sensitivity function was used to complete the long wavelength tail of the tsetse sensitivity curve from 626-700 nm, with an adjoining one-way spline interpolation between the 6 data points either side of the gap using SRS1 Cubic Splines for Excel (SRS1 Software LLC., Boston MA, USA).

**2.3 Tsetse R7y sensitivity function**

The spectral sensitivity of the *Glossina* R7y function was well-matched to that for *Musca* at the short wavelength end, but differed in terms of an enhanced shoulder of sensitivity in the blue region of the spectrum [5]. Like R1-6, the sensitivity of R7y has been measured at much finer wavelength increments for *Musca*, exposing a distinct fine structure due to a sensitising pigment [3,4]. As such, a tsetse spectral sensitivity function was constructed by using the generic function at the short wavelength end, switching to the extracted tsetse function where the two diverged. Tsetse data were available up to ca. 498 nm because only post-feeding data were available at the longer wavelength, and using these would have affected the weighted mean.

Completion of the long wavelength tail of the tsetse function was guided by modelling of its sensitivity as above (equations 1 and 2). For R7y, a λmax = 430 nm rhodopsin alpha band nomogram was used [9], *l* = 130 μm, and *d* = 1 μm [6]. The spectral sensitivity function calculated in this way for the active photopigment (*ap*) was modified to account for the presence of a photostable carotenoid screening pigment (*pp*) also located in the R7y rhabdom, according to [7]:

(3) $S_{i}(\lambda)=(\frac{\gamma_{ap}}{\gamma_{ap}+\gamma_{pp}})\cdot(1-\exp^{-(\gamma_{ap}+\gamma_{pp})})$

Where *γ* represents *α(λ)∙c∙l∙η(λ)* from equation (1). For the photostable screening pigment, *α(λ)∙η(λ)* was approximated by extracting the direct measurements in figures 9a and 9b (curve 3) of [13], and computing an average. I set *c* for the photostable screening pigment to 0.023, providing *c∙l* = 2.99 (equivalent to screening from R7y on R8y, as computed by [7]), or 0, mimicking a complete absence of screening pigment.

S_R7y_(λ) was first computed with photostable pigment *c* = 0.023, and scaled to match the relative sensitivity of the blue shoulder in the normalised generic curve. Photostable pigment *c* was then set to zero, and the resulting curve was well-matched to the portion >400 nm of the tsetse curve. These calculated values were used to complete the long wavelength tail of the tsetse sensitivity function from 524-700 nm, with the gap from 500-522 nm filled by one-way spline interpolation between the six values either side of the missing portion.

**2.4 Generic and tsetse-specific R8y sensitivity functions**

In order to extrapolate the long wavelength tail of the existing *Musca* R8y sensitivity function [1,3,4], a similar approach was followed as for R7y. *S_R8y_(λ)* was first calculated using equation (1), based upon λmax = 520 nm nomogram [9], and *l* = 70 μm [6,8]. This sensitivity function was then modified for screening by the overlying R7y rhabdom following [7]:

(4) $S'_{R8}(\lambda)=\exp^{-\gamma_{R7}\cdot c\cdot l}\cdot S_{R8}(\lambda)$

Here, *γ_R7_* represents the measured extinction of R7y extracted from figure 1 of [13]. Following [7], *c∙l* was set to 3.0. The resulting function was then normalised to its peak value and used to complete the long wavelength tail of the published sensitivity function from 626 to 700 nm. The intervening gap from 600-626 nm was completed by one-way spline interpolation using the 6 data points either side of the gap.

In the construction of the tsetse R8y sensitivity function, the assumption was that the removal of screening pigment from the overlying R7y rhabdom would enhance the sensitivity of R8y at >400 nm, but not affect screening at shorter wavelengths. Initially, an R8y sensitivity function was constructed as for *Musca* using equation (4), but the product *c∙l* for R7y screening was reduced to 0.3 (1/10^th^). The resulting normalised curve had enhanced sensitivity in the blue region of the spectrum and was a relatively good match to the experimental data for tsetse [5]. This function was used to provide a long wavelength tail for the tsetse data extracted from [5] between 626-700 nm, with the gap between functions filled by one-way spline interpolation using 6 data points either side of the gap. At the short wavelength end, the finer resolution spectral sensitivity measurements for the sensitising pigment from *Musca* were employed. To reflect the enhanced sensitivity of the unscreened tsetse R8y receptor, and thus the reduced relative height of the sensitising pigment peak, the generic function was rescaled to 90% of its original height. The appropriate re-scaling was determined by the ratio of the screened to unscreened R8y calculated sensitivity peaks before normalisation. Re-scaled *Musca* data were thus duplicated in the tsetse function at short wavelengths, transferring to the tsetse data where the functions diverged.

**References**

1. Santer RD (2014) A colour opponent model that explains tsetse fly attraction to visual baits and can be used to investigate more efficacious bait materials. PLoS Negl Trop Dis 8: e3360.

2. Santer RD (2015) A receptor-based explanation for tsetse fly catch distribution between coloured cloth panels and flanking nets. PLoS Negl Trop Dis 9: e0004121.

3. Hardie RC (1986) The photoreceptor array of the dipteran retina. Trends Neurosci 9: 419-423.

4. Hardie RC, Kirschfeld K (1983) Ultraviolet sensitivity of fly photoreceptors R7 and R8: Evidence for a sensitising function. Biophys Struct Mech 9: 171-180.

5. Hardie RC, Vogt K, Rudolph A (1989) The compound eye of the tsetse fly (*Glossina morsitans morsitans* and *Glossina palpalis palpalis*). J Insect Physiol 35: 423-431.

6. Snyder AW, Pask C (1973) Spectral sensitivity of dipteran retinula cells. J Comp Physiol 84: 59-76.

7. Hardie RC, Franceschini N, McIntyre PD (1979) Electrophysiological analysis of fly retina II. Spectral and polarisation sensitivity in R7 and R8. J Comp Physiol 133: 23-29.

8. Stavenga DG, Oberwinkler J, Postma M (2000) Modelling primary visual processes in insect photoreceptors. In: Stavenga DG, DeGrip WJ, Pugh Jr. EN, editors. Handbook of Biological Physics, volume 3. Amsterdam: Elsevier.

9. Stavenga DG, Smits RP, Hoenders BJ (1993) Simple exponential functions describing the absorbance bands of visual pigment spectra. Vision Res 33: 1011-1017.

10. Tummers B (2006) DataThief III <[http://datathief.org/>](http://datathief.org/%3e).

11. Smakman JGJ, Stavenga DG (1986) Spectral sensitivity of blowfly photoreceptors: dependence on waveguide effects and pigment concentration. Vision Res 26: 1019-1025.

12. Beersma DGM, Hoenders BJ, Huiser AMJ, van Torn P (1982) Refractive index of the fly rhabdomere. J Opt Soc Am 72: 583-588.

13. McIntyre P, Kirschfeld K (1981) Absoption properties of a photostable pigment (P456) in rhabdomere 7 of the fly. J Comp Physiol 143: 3-15.
