# Supplementary material for: Developing photoreceptor-based models of visual attraction in riverine tsetse, for use in the engineering of more-attractive polyester fabrics for control devices
Source: PLoS Negl Trop Dis. 2017 Mar 17;11(3):e0005448. doi: 10.1371/journal.pntd.0005448 (PMC5371378; doi:10.1371/journal.pntd.0005448)
Supplement: S2 Text — A full description of model selection procedures and results. (DOCX) [file pntd.0005448.s003.docx]

**S2 Text. Supplementary results: Model selection**

**1. Model selection methods**

The approach taken in this study was to evaluate a series of GEE models that used calculated photoreceptor excitation values, or indices constructed from them, to predict the total combined catches of 37 tiny targets recorded in a field study [1].

For each model, the SPSS GENLIN algorithm used to implement the GEE provided the corrected quasi-likelihood under independence model criterion, QICC, which is a modification of AIC (Akaike’s Information Criterion) for use with GEE that is corrected for small sample sizes with a stricter penalty for model complexity [2,3,4]. Lower values of QICC indicate better fit to the data [3]. At each analysis stage, ΔQICC values were calculated for each candidate model under consideration [5]:

$$\Delta QICC_{i}= QICC_{i} - QICC_{\min}$$

Models with ΔQICC <2 were deemed competitive with the best model [5]. In addition, Akaike weights, *w_i_*, were computed to indicate relative weight of evidence supporting an individual model as being the best approximating model within a set of *R* competitive models (on the assumption that one of them is indeed the best approximating model) [5]:

$$w_{i}= \frac{\exp(-\frac{1}{2}\Delta QICC_{i})}{\sum_{r=1}^{R} \exp(-\frac{1}{2}\Delta QICC_{r})}$$

Note that, therefore, Akaike weights can be compared within a set of models that use the same tsetse catch data (e.g. that for males or females), and the same photoreceptor excitation values (e.g. those from screened or unscreened sensitivities), presented in the same table. They cannot be directly compared with models in different analysis blocks or results tables.

**2. Models based upon weighted photoreceptor excitations**

In the first stage of analysis a set of GEE models comprising every possible combination of between one and five of the calculated photoreceptor excitation values as predictors was evaluated. This was conducted separately for the male and female catch datasets, and for the assumptions of screened (Fig 1B) and unscreened (Fig 1C) photoreceptor sensitivities.

Under the assumption of both screened and unscreened sensitivities, more complex models provided better explanations for tsetse attraction, and several models were deemed to have strong support via low ΔQICC and high *w_i_* (Tables A and B). When screened sensitivities were assumed, of the three models with strongest support only one was common to the male and female datasets, and that employed the excitations of R7y, R7p, and R8y as predictors (Table A). When unscreened sensitivities were assumed, there was only moderate support for the R7y, R7p, R8y model and other three-predictor models that included the excitations of the three implicated photoreceptors. The most strongly supported models were more complex (Table B). Of these, the best-supported model common to the male and female datasets included the excitations of R1-6, R7p, R8p, and R8y (Table B).

**Table A: Comparison of tsetse attraction models based upon photoreceptor excitation values calculated using screened sensitivity functions.**

|  | **A. Males** (k=0.054) | | | **B. Females** (k=0.065) | | |
| --- | --- | --- | --- | --- | --- | --- |
|  | QICC | ΔQICC | *w_i_* | QICC | ΔQICC | *w_i_* |
| Intercept only | 175.606 | 88.537 | *0.000* | 191.834 | 105.982 | *0.000* |
| **1 photoreceptor models** |  |  |  |  |  |  |
| E_R1-6_ | 168.220 | 81.151 | *0.000* | 188.741 | 102.889 | *0.000* |
| E_R7p_ | 109.321 | 22.252 | *0.000* | 116.470 | 30.618 | *0.000* |
| E_R7y_ | 168.401 | 81.332 | *0.000* | 188.153 | 102.301 | *0.000* |
| E_R8p_ | 177.351 | 90.282 | *0.000* | 193.668 | 107.816 | *0.000* |
| E_R8y_ | 139.157 | 52.088 | *0.000* | 161.126 | 75.274 | *0.000* |
| **2 photoreceptor models** |  |  |  |  |  |  |
| E_R1-6_, E_R7p_ | 111.201 | 24.132 | *0.000* | 115.075 | 29.223 | *0.000* |
| E_R1-6_, E_R7y_ | 169.856 | 82.787 | *0.000* | 191.507 | 105.655 | *0.000* |
| E_R1-6_, E_R8p_ | 111.313 | 24.244 | *0.000* | 121.610 | 35.758 | *0.000* |
| E_R1-6_, E_R8y_ | 108.938 | 21.869 | *0.000* | 119.413 | 33.561 | *0.000* |
| E_R7p_, E_R7y_ | 102.994 | 15.925 | *0.000* | 101.033 | 15.181 | *0.000* |
| E_R7p_, E_R8p_ | 107.342 | 20.273 | *0.000* | 108.694 | 22.842 | *0.000* |
| E_R7p_, E_R8y_ | 108.733 | 21.664 | *0.000* | 118.104 | 32.252 | *0.000* |
| E_R7y_, E_R8p_ | 143.890 | 56.821 | *0.000* | 158.002 | 72.150 | *0.000* |
| E_R7y_, E_R8y_ | 135.508 | 48.439 | *0.000* | 156.035 | 70.183 | *0.000* |
| E_R8p_, E_R8y_ | 108.859 | 21.790 | *0.000* | 119.189 | 33.337 | *0.000* |
| **3 photoreceptor models** |  |  |  |  |  |  |
| E_R1-6_, E_R7p_, E_R7y_ | 88.907 | 1.838 | *0.080* | 85.852 | 0.000 | ***0.217*** |
| E_R1-6_, E_R7p_, E_R8p_ | 91.719 | 4.650 | *0.020* | 94.607 | 8.755 | *0.003* |
| E_R1-6_, E_R7p_, E_R8y_ | 87.197 | 0.128 | ***0.188*** | 89.298 | 3.446 | *0.039* |
| E_R1-6_, E_R7y_, E_R8p_ | 100.066 | 12.997 | *0.000* | 108.976 | 23.124 | *0.000* |
| E_R1-6_, E_R7y_, E_R8y_ | 92.207 | 5.138 | *0.015* | 101.113 | 15.261 | *0.000* |
| E_R1-6_, E_R8p_, E_R8y_ | 110.914 | 23.845 | *0.000* | 121.146 | 35.294 | *0.000* |
| E_R7p_, E_R7y_, E_R8p_ | 94.430 | 7.361 | *0.005* | 88.287 | 2.435 | *0.064* |
| E_R7p_, E_R7y_, E_R8y_ | 87.069 | 0.000 | ***0.200*** | 85.951 | 0.099 | ***0.207*** |
| E_R7p_, E_R8p_, E_R8y_ | 88.533 | 1.464 | *0.096* | 91.107 | 5.255 | *0.016* |
| E_R7y_, E_R8p_, E_R8y_ | 95.082 | 8.013 | *0.004* | 103.707 | 17.855 | *0.000* |
| **4 photoreceptor models** |  |  |  |  |  |  |
| E_R1-6_, E_R7p_, E_R7y_, E_R8p_ | 90.781 | 3.712 | *0.031* | 87.539 | 1.687 | *0.094* |
| E_R1-6_, E_R7p_, E_R7y_, E_R8y_ | 88.883 | 1.814 | *0.081* | 87.698 | 1.846 | *0.086* |
| E_R1-6_, E_R7p_, E_R8p_, E_R8y_ | 87.897 | 0.828 | ***0.132*** | 90.028 | 4.176 | *0.027* |
| E_R1-6_, E_R7y_, E_R8p_, E_R8y_ | 93.969 | 6.900 | *0.006* | 103.180 | 17.328 | *0.000* |
| E_R7p_, E_R7y_, E_R8p_, E_R8y_ | 89.056 | 1.987 | *0.074* | 86.585 | 0.733 | ***0.151*** |
| **5 photoreceptor model** |  |  |  |  |  |  |
| E_R1-6_, E_R7p_, E_R7y_, E_R8p_, E_R8y_ | 89.280 | 2.211 | *0.066* | 87.476 | 1.624 | *0.096* |

*Spectral sensitivity functions shown in Fig 1B; k is the dispersion parameter of the negative binomial distribution estimated for the saturated model. Lower QICC and ΔQICC indicate better fit to the data. Akaike weights (w_i_) quantify relative weight of evidence for each model in the table. Strongest supported models indicated by bold type; that common to males and females underlined.*

**Table B: Comparison of tsetse attraction models based upon photoreceptor excitation values calculated using unscreened sensitivity functions.**

|  | **A. Males** (k=0.050) | | | **B. Females** (k=0.060) | | |
| --- | --- | --- | --- | --- | --- | --- |
|  | QICC | ΔQICC | *w_i_* | QICC | ΔQICC | *w_i_* |
| Intercept only | 187.498 | 99.443 | *0.000* | 205.877 | 119.528 | *0.000* |
| **1 photoreceptor models** |  |  |  |  |  |  |
| E_R1-6_ | 179.493 | 91.438 | *0.000* | 202.420 | 116.071 | *0.000* |
| E_R7p_ | 116.523 | 28.468 | *0.000* | 124.775 | 38.426 | *0.000* |
| E_R7y_ | 187.104 | 99.049 | *0.000* | 207.450 | 121.101 | *0.000* |
| E_R8p_ | 189.226 | 101.171 | *0.000* | 207.699 | 121.350 | *0.000* |
| E_R8y_ | 172.194 | 84.139 | *0.000* | 196.341 | 109.992 | *0.000* |
| **2 photoreceptor models** |  |  |  |  |  |  |
| E_R1-6_, E_R7p_ | 118.376 | 30.321 | *0.000* | 123.086 | 36.737 | *0.000* |
| E_R1-6_, E_R7y_ | 163.787 | 75.732 | *0.000* | 184.901 | 98.552 | *0.000* |
| E_R1-6_, E_R8p_ | 118.494 | 30.439 | *0.000* | 130.129 | 43.780 | *0.000* |
| E_R1-6_, E_R8y_ | 144.947 | 56.892 | *0.000* | 164.105 | 77.756 | *0.000* |
| E_R7p_, E_R7y_ | 111.018 | 22.963 | *0.000* | 110.611 | 24.262 | *0.000* |
| E_R7p_, E_R8p_ | 114.236 | 26.181 | *0.000* | 116.218 | 29.869 | *0.000* |
| E_R7p_, E_R8y_ | 119.043 | 30.988 | *0.000* | 126.407 | 40.058 | *0.000* |
| E_R7y_, E_R8p_ | 171.986 | 83.931 | *0.000* | 192.761 | 106.412 | *0.000* |
| E_R7y_, E_R8y_ | 156.491 | 68.436 | *0.000* | 177.419 | 91.070 | *0.000* |
| E_R8p_, E_R8y_ | 127.147 | 39.092 | *0.000* | 140.494 | 54.145 | *0.000* |
| **3 photoreceptor models** |  |  |  |  |  |  |
| E_R1-6_, E_R7p_, E_R7y_ | 93.658 | 5.603 | *0.026* | 92.307 | 5.958 | *0.026* |
| E_R1-6_, E_R7p_, E_R8p_ | 97.404 | 9.349 | *0.004* | 100.952 | 14.603 | *0.000* |
| E_R1-6_, E_R7p_, E_R8y_ | 90.145 | 2.090 | ***0.150*** | 90.738 | 4.389 | ***0.058*** |
| E_R1-6_, E_R7y_, E_R8p_ | 109.673 | 21.618 | *0.000* | 121.080 | 34.731 | *0.000* |
| E_R1-6_, E_R7y_, E_R8y_ | 113.239 | 25.184 | *0.000* | 132.158 | 45.809 | *0.000* |
| E_R1-6_, E_R8p_, E_R8y_ | 115.804 | 27.749 | *0.000* | 126.691 | 40.342 | *0.000* |
| E_R7p_, E_R7y_, E_R8p_ | 97.089 | 9.034 | *0.005* | 90.584 | 4.235 | *0.063* |
| E_R7p_, E_R7y_, E_R8y_ | 92.411 | 4.356 | *0.048* | 91.590 | 5.241 | *0.038* |
| E_R7p_, E_R8p_, E_R8y_ | 94.321 | 6.266 | *0.019* | 97.030 | 10.681 | *0.002* |
| E_R7y_, E_R8p_, E_R8y_ | 107.394 | 19.339 | *0.000* | 119.311 | 32.962 | *0.000* |
| **4 photoreceptor models** |  |  |  |  |  |  |
| E_R1-6_, E_R7p_, E_R7y_, E_R8p_ | 95.330 | 7.275 | *0.011* | 91.435 | 5.086 | *0.041* |
| E_R1-6_, E_R7p_, E_R7y_, E_R8y_ | 91.047 | 2.992 | *0.096* | 93.239 | 6.890 | *0.017* |
| E_R1-6_, E_R7p_, E_R8p_, E_R8y_ | 88.055 | 0.000 | ***0.426*** | 86.349 | 0.000 | ***0.521*** |
| E_R1-6_, E_R7y_, E_R8p_, E_R8y_ | 106.068 | 18.013 | *0.000* | 120.242 | 33.893 | *0.000* |
| E_R7p_, E_R7y_, E_R8p_, E_R8y_ | 94.151 | 6.096 | *0.020* | 90.772 | 4.423 | *0.057* |
| **5 photoreceptor model** |  |  |  |  |  |  |
| E_R1-6_, E_R7p_, E_R7y_, E_R8p_, E_R8y_ | 89.620 | 1.565 | ***0.195*** | 88.511 | 2.162 | ***0.177*** |

*Spectral sensitivity functions shown in Fig 1C; Other conventions as in Table A.*

**3. Alternative models of fly colour vision**

In order to represent the categorical encoding of colour within separate opponent mechanisms for the ‘y’ and ‘p’ type ommatidia (c.f. [6]), categorical predictors were calculated for each ommatidium type. These relate to the *Opp_s_* index (E_R7_*_s_*/E_R8_*_s_*) reported in main text as follows:

$$Cat_{s}(Opp_{s})\left\{\begin{matrix} 0 if Opp_{s}<1 \\ 1 otherwise \end{matrix}\right.$$

Here, *s* denotes the ‘y’ or ‘p’ opponent systems. In addition, the resulting four colour categories (Cat_y_=1, Cat_p_=1; Cat_y_=1, Cat_p_=0; Cat_y_=0, Cat_p_=1; and Cat_y_=0, Cat_p_=0) were entered into the analysis as separate nominal variables, and Cat_y&p_ is used as shorthand for this set.

Models using categorical predictors for ‘y’ and ‘p’ opponent systems provided poor explanations for combined catch data for the male and female datasets, and for analyses based on screened and unscreened photoreceptor sensitivity functions (Table C). In all cases, the addition of the E_R1-6_ predictor to represent the separate evaluation of luminance substantially improved model fit to the data (indicated by a decrease in QICC and ΔQICC). Despite this, none of these models was competitive with the previously described weighted photoreceptor excitation models when evaluated using ΔQICC or *w_i_* (Table C).

**Table C: Evaluation of models explaining tsetse attraction using categorical colour predictors, with or without excitation of R1-6 as an additional predictor.**

|  | **A. Males** | | | **B. Females** | | |
| --- | --- | --- | --- | --- | --- | --- |
| *(i) Screened sensitivities* | (k=0.054) | | | (k=0.065) | | |
|  | QICC | ΔQICC | *w_i_* | QICC | ΔQICC | *w_i_* |
| E_R7p_, E_R7y_, E_R8y_ | 87.069 | 0.000 | **0.502** | 85.951 | 0.000 | **0.626** |
| E_R1-6_, E_R7p_, E_R8p_, E_R8y_ | 87.897 | 0.828 | **0.332** | 90.028 | 4.077 | **0.082** |
| E_R1-6_, E_R7p_, E_R7y_, E_R8p_, E_R8y_ | 89.280 | 2.211 | **0.166** | 87.476 | 1.525 | **0.292** |
|  |  |  |  |  |  |  |
| Cat_y_, Cat_p_ | 161.366 | 74.297 | 0.000 | 162.665 | 76.714 | 0.000 |
| Cat_y_, Cat_p_, E_R1-6_ | 135.044 | 47.975 | 0.000 | 138.518 | 52.567 | 0.000 |
| Cat_y&p_ | 160.797 | 73.728 | 0.000 | 165.203 | 79.252 | 0.000 |
| Cat_y&p_, E_R1-6_ | 136.647 | 49.578 | 0.000 | 143.056 | 57.105 | 0.000 |
| *(ii) Unscreened sensitivities* | (k=0.050) | | | (k=0.060) | | |
|  | QICC | ΔQICC | *w_i_* | QICC | ΔQICC | *w_i_* |
| E_R7p_, E_R7y_, E_R8y_ | 92.411 | 4.356 | **0.072** | 91.590 | 5.241 | **0.052** |
| E_R1-6_, E_R7p_, E_R8p_, E_R8y_ | 88.055 | 0.000 | **0.637** | 86.349 | 0.000 | **0.708** |
| E_R1-6_, E_R7p_, E_R7y_, E_R8p_, E_R8y_ | 89.620 | 1.565 | **0.291** | 88.511 | 2.162 | **0.240** |
|  |  |  |  |  |  |  |
| Cat_y_, Cat_p_ | 173.144 | 85.089 | 0.000 | 175.286 | 88.937 | 0.000 |
| Cat_y_, Cat_p_, E_R1-6_ | 145.070 | 57.015 | 0.000 | 149.657 | 63.308 | 0.000 |
| Cat_y&p_ | 173.933 | 85.878 | 0.000 | 177.357 | 91.008 | 0.000 |
| Cat_y&p_, E_R1-6_ | 147.150 | 59.095 | 0.000 | 152.503 | 66.154 | 0.000 |

*Conventions as in Table A.*

A range of additional models were considered in which predictors were computed from R7 and R8 photoreceptor excitations to represent the evaluation of colour information separated from luminance, but without categorical coding of their outputs. For all such models, the fit to the data was poor unless the E_R1-6_ photoreceptor response was added in representation of a separate luminance channel (Table D). Based upon ΔQICC and *w_i_*, the fit of these models to the data was improved versus the earlier-generated weighted photoreceptor excitation models and colour categorical models, for male and female datasets and under the assumption of either screened or unscreened photoreceptor sensitivities (Table D). For the male dataset, a model based upon ‘y’ and ‘p’ opponent systems was strongest supported, although there was also strong support for the generic colour model (Table D). For the female dataset that pattern was reversed, with the generic colour model the strongest supported (Table D).

Note that despite the identification of best-supported models in this analysis several other candidate models could not be ruled out and a degree of variation in the ‘best’ model would be expected from sample to sample. This variation is perhaps not surprising given that excitations calculated for different photoreceptor types were often strongly correlated across the complete set of fabrics examined (Tables 1 and 2).

**Table D: Evaluation of models explaining tsetse attraction using R7-8 photoreceptor excitations combined into two opponent channels, or represented relative to one another, with or without excitation of R1-6 as an additional predictor.**

|  | **A. Males** | | | **B. Females** | | |
| --- | --- | --- | --- | --- | --- | --- |
| *(i) Screened sensitivities* | (k=0.054) | | | (k=0.065) | | |
|  | QICC | ΔQICC | *w_i_* | QICC | ΔQICC | *w_i_* |
| E_R7p_, E_R7y_, E_R8y_ | 87.069 | 8.785 | 0.007 | 85.951 | 10.165 | 0.004 |
| E_R1-6_, E_R7p_, E_R8p_, E_R8y_ | 87.897 | 9.613 | 0.004 | 90.028 | 14.242 | 0.001 |
| E_R1-6_, E_R7p_, E_R7y_, E_R8p_, E_R8y_ | 89.280 | 10.996 | 0.002 | 87.476 | 11.690 | 0.002 |
|  |  |  |  |  |  |  |
| [E_R7y_-E_R8y_], [E_R7p_-E_R8p_] | 122.441 | 44.157 | 0.000 | 120.765 | 44.979 | 0.000 |
| [E_R7y_-E_R8y_], [E_R7p_-E_R8p_], E_R1-6_ | 88.777 | 10.493 | 0.003 | 89.694 | 13.908 | 0.001 |
| [E_R7y_/(E_R7y_+E_R8y_)], [E_R7p_/(E_R7p_+E_R8p_)] | 101.596 | 23.312 | 0.000 | 101.633 | 25.847 | 0.000 |
| [E_R7y_/(E_R7y_+E_R8y_)], [E_R7p_/(E_R7p_+E_R8p_)], E_R1-6_ | 80.365 | 2.081 | **0.193** | 79.526 | 3.740 | **0.110** |
| Opp_y_, Opp_p_ | 110.865 | 32.581 | 0.000 | 112.165 | 36.379 | 0.000 |
| Opp_y_, Opp_p_, E_R1-6_ | 78.284 | 0.000 | **0.548** | 78.620 | 2.834 | **0.172** |
|  |  |  |  |  |  |  |
| Rel_R7p_, Rel_R7y_, Rel_R8p_, Rel_R8y_ | 102.922 | 24.638 | 0.000 | 99.066 | 23.280 | 0.000 |
| Rel_R7p_, Rel_R7y_, Rel_R8p_, Rel_R8y_, E_R1-6_ | 79.913 | 1.629 | **0.243** | 75.786 | 0.000 | **0.711** |
| *(ii) Unscreened sensitivities* | (k=0.050) | | | (k=0.060) | | |
|  | QICC | ΔQICC | *w_i_* | QICC | ΔQICC | *w_i_* |
| E_R7p_, E_R7y_, E_R8y_ | 92.411 | 9.233 | 0.005 | 91.590 | 11.705 | 0.002 |
| E_R1-6_, E_R7p_, E_R8p_, E_R8y_ | 88.055 | 4.877 | 0.042 | 86.349 | 6.464 | 0.031 |
| E_R1-6_, E_R7p_, E_R7y_, E_R8p_, E_R8y_ | 89.620 | 6.442 | 0.019 | 88.511 | 8.626 | 0.010 |
|  |  |  |  |  |  |  |
| [E_R7y_-E_R8y_], [E_R7p_-E_R8p_] | 130.938 | 47.760 | 0.000 | 129.101 | 49.216 | 0.000 |
| [E_R7y_-E_R8y_], [E_R7p_-E_R8p_], E_R1-6_ | 94.243 | 11.065 | 0.002 | 95.462 | 15.577 | 0.000 |
| [E_R7y_/(E_R7y_+E_R8y_)], [E_R7p_/(E_R7p_+E_R8p_)] | 112.705 | 29.527 | 0.000 | 110.853 | 30.968 | 0.000 |
| [E_R7y_/(E_R7y_+E_R8y_)], [E_R7p_/(E_R7p_+E_R8p_)], E_R1-6_ | 85.402 | 2.224 | **0.159** | 84.529 | 4.644 | **0.076** |
| Opp_y_, Opp_p_ | 126.400 | 43.222 | 0.000 | 125.860 | 45.975 | 0.000 |
| Opp_y_, Opp_p_, E_R1-6_ | 83.178 | 0.000 | **0.484** | 83.823 | 3.938 | **0.108** |
|  |  |  |  |  |  |  |
| Rel_R7p_, Rel_R7y_, Rel_R8p_, Rel_R8y_ | 113.258 | 30.080 | 0.000 | 107.272 | 27.387 | 0.000 |
| Rel_R7p_, Rel_R7y_, Rel_R8p_, Rel_R8y_, E_R1-6_ | 84.217 | 1.039 | **0.288** | 79.885 | 0.000 | **0.773** |

*Conventions as in Table A. Opp_s_ and Rel_r_ indices are as defined in main text; predictors in square brackets are alternative computations to represent processing of photoreceptor responses within ‘y’ and ‘p’ opponent systems.*

**4. Robustness of model selection to the assumed R1-6 sensitivity function**

The model selection procedures described above used an R1-6 sensitivity function based on that recorded electrophysiologically from *G. m. morsitans*, regardless of whether screened or unscreened functions for R7-8 were used. Model selection was also conducted using screened R7-8 sensitivity functions (Fig 1B) and the R1-6 sensitivity function characteristic of *Musca* and *Calliphora* (Fig 1A). Model selection in this way yielded similar results to the analyses presented above using screened photoreceptor sensitivities (Table E).

**Table E. Evaluation of photoreceptor-based models of tsetse attraction assuming screened R7-8 sensitivity functions (Fig 1B) and generic R1-6 sensitivity function (Fig 1A).**

|  | **A. Males** (k=0.054) | | | **B. Females** (k=0.060) | | |
| --- | --- | --- | --- | --- | --- | --- |
|  | QICC | ΔQICC | w_i_ | QICC | ΔQICC | w_i_ |
| Intercept only | 175.606 | 98.460 | 0.000 | 205.877 | 124.385 | 0.000 |
| **1 photoreceptor models** |  |  |  |  |  |  |
| E_R1-6_ | 172.159 | 95.013 | 0.000 | 205.651 | 124.159 | 0.000 |
| E_R7p_ | 109.321 | 32.175 | 0.000 | 124.775 | 43.283 | 0.000 |
| E_R7y_ | 168.401 | 91.255 | 0.000 | 201.788 | 120.296 | 0.000 |
| E_R8p_ | 177.351 | 100.205 | 0.000 | 207.699 | 126.207 | 0.000 |
| E_R8y_ | 139.157 | 62.011 | 0.000 | 172.809 | 91.317 | 0.000 |
| **2 photoreceptor models** |  |  |  |  |  |  |
| E_R1-6_, E_R7p_ | 110.217 | 33.071 | 0.000 | 120.858 | 39.366 | 0.000 |
| E_R1-6_, E_R7y_ | 169.355 | 92.209 | 0.000 | 203.726 | 122.234 | 0.000 |
| E_R1-6_, E_R8p_ | 110.127 | 32.981 | 0.000 | 130.974 | 49.482 | 0.000 |
| E_R1-6_, E_R8y_ | 109.101 | 31.955 | 0.000 | 127.379 | 45.887 | 0.000 |
| E_R7p_, E_R7y_ | 102.994 | 25.848 | 0.000 | 107.990 | 26.498 | 0.000 |
| E_R7p_, E_R8p_ | 107.342 | 30.196 | 0.000 | 116.218 | 34.726 | 0.000 |
| E_R7p_, E_R8y_ | 108.733 | 31.587 | 0.000 | 126.398 | 44.906 | 0.000 |
| E_R7y_, E_R8p_ | 143.890 | 66.744 | 0.000 | 169.166 | 87.674 | 0.000 |
| E_R7y_, E_R8y_ | 135.508 | 58.362 | 0.000 | 167.159 | 85.667 | 0.000 |
| E_R8p_, E_R8y_ | 108.859 | 31.713 | 0.000 | 127.533 | 46.041 | 0.000 |
| **3 photoreceptor models** |  |  |  |  |  |  |
| E_R1-6_, E_R7p_, E_R7y_ | 89.770 | 12.624 | 0.001 | 92.512 | 11.020 | 0.002 |
| E_R1-6_, E_R7p_, E_R8p_ | 91.928 | 14.782 | 0.000 | 102.641 | 21.149 | 0.000 |
| E_R1-6_, E_R7p_, E_R8y_ | 87.550 | 10.404 | **0.004** | 95.161 | 13.669 | 0.001 |
| E_R1-6_, E_R7y_, E_R8p_ | 99.410 | 22.264 | 0.000 | 117.906 | 36.414 | 0.000 |
| E_R1-6_, E_R7y_, E_R8y_ | 93.540 | 16.394 | 0.000 | 107.493 | 26.001 | 0.000 |
| E_R1-6_, E_R8p_, E_R8y_ | 110.816 | 33.670 | 0.000 | 129.829 | 48.337 | 0.000 |
| E_R7p_, E_R7y_, E_R8p_ | 94.430 | 17.284 | 0.000 | 94.173 | 12.681 | 0.001 |
| E_R7p_, E_R7y_, E_R8y_ | 87.069 | 9.923 | **0.005** | 91.658 | 10.166 | **0.004** |
| E_R7p_, E_R8p_, E_R8y_ | 88.533 | 11.387 | 0.002 | 97.198 | 15.706 | 0.000 |
| E_R7y_, E_R8p_, E_R8y_ | 95.082 | 17.936 | 0.000 | 110.735 | 29.243 | 0.000 |
| **4 photoreceptor models** |  |  |  |  |  |  |
| E_R1-6_, E_R7p_, E_R7y_, E_R8p_ | 91.279 | 14.133 | 0.001 | 94.477 | 12.985 | 0.001 |
| E_R1-6_, E_R7p_, E_R7y_, E_R8y_ | 88.962 | 11.816 | 0.002 | 93.457 | 11.965 | 0.001 |
| E_R1-6_, E_R7p_, E_R8p_, E_R8y_ | 87.960 | 10.814 | **0.003** | 92.279 | 10.787 | **0.003** |
| E_R1-6_, E_R7y_, E_R8p_, E_R8y_ | 95.293 | 18.147 | 0.000 | 108.401 | 26.909 | 0.000 |
| E_R7p_, E_R7y_, E_R8p_, E_R8y_ | 89.056 | 11.910 | 0.002 | 92.192 | 10.700 | 0.003 |
| **5 photoreceptor model** |  |  |  |  |  |  |
| E_R1-6_, E_R7p_, E_R7y_, E_R8p_, E_R8y_ | 88.890 | 11.744 | 0.002 | 88.859 | 7.367 | **0.015** |
| **Categorical colour models** |  |  |  |  |  |  |
| Cat_y_, Cat_p_ | 161.366 | 84.220 | 0.000 | 174.254 | 92.762 | 0.000 |
| Cat_y_, Cat_p_, E_R1-6_ | 140.325 | 63.179 | 0.000 | 153.768 | 72.276 | 0.000 |
| Cat_y&p_ | 160.797 | 83.651 | 0.000 | 176.837 | 95.345 | 0.000 |
| Cat_y&p_, E_R1-6_ | 141.732 | 64.586 | 0.000 | 158.480 | 76.988 | 0.000 |
| **Colour system models** |  |  |  |  |  |  |
| [E_R7y_-E_R8y_], [E_R7p_-E_R8p_] | 122.441 | 45.295 | 0.000 | 129.188 | 47.696 | 0.000 |
| [E_R7y_-E_R8y_], [E_R7p_-E_R8p_], E_R1-6_ | 88.286 | 11.140 | 0.002 | 95.529 | 14.037 | 0.001 |
| [E_R7y_/(E_R7y_+E_R8y_)], [E_R7p_/(E_R7p_+E_R8p_)] | 101.596 | 24.450 | 0.000 | 108.610 | 27.118 | 0.000 |
| [E_R7y_/(E_R7y_+E_R8y_)], [E_R7p_/(E_R7p_+E_R8p_)], E_R1-6_ | 79.927 | 2.781 | **0.161** | 84.768 | 3.276 | **0.113** |
| Opp_y_, Opp_p_ | 110.865 | 33.719 | 0.000 | 119.928 | 38.436 | 0.000 |
| Opp_y_, Opp_p_, E_R1-6_ | 77.146 | 0.000 | **0.646** | 82.987 | 1.495 | **0.275** |
| Rel_R7p_, Rel_R7y_, Rel_R8p_, Rel_R8y_ | 102.922 | 25.776 | 0.000 | 105.701 | 24.209 | 0.000 |
| Rel_R7p_, Rel_R7y_, Rel_R8p_, Rel_R8y_, E_R1-6_ | 79.821 | 2.675 | **0.170** | 81.492 | 0.000 | **0.581** |

*Spectral sensitivity functions shown in Fig 1A and 1B; Conventions as in Table A.*

**References**

1. Lindh JM, Goswami P, Blackburn R, Arnold SEJ, Vale GA, et al. (2012) Optimizing the colour and fabric of targets for the control of the tsetse fly *Glossina fuscipes fuscipes*. PLoS Negl Trop Dis 6: e1661.

2. Pan W (2001) Akaike's information criterion in generalized estimating equations. Biometrics 57: 120-125.

3. Garson GD (2012) Generalized linear models and generalized estimating equations. Asheboro, N.C.: Statistical Associates Publishing.

4. Hardin JW, Hilbe JM (2012) Generalized Estimating Equations, second edition. Boca Raton: CRC Press.

5. Burnham KP, Anderson DR (2002) Model Selection and Multimodel Inference: A Practical Information-Theoretic Approach, second edition. New York: Springer-Verlag.

6. Troje N (1993) Spectral categories in the learning behaviour of blowflies. Z Naturforsch 48: 96-104.
