# Supplementary figures and images for: Developing photoreceptor-based models of visual attraction in riverine tsetse, for use in the engineering of more-attractive polyester fabrics for control devices
Source: PLoS Negl Trop Dis. 2017 Mar 17;11(3):e0005448. doi: 10.1371/journal.pntd.0005448 (PMC5371378; doi:10.1371/journal.pntd.0005448)

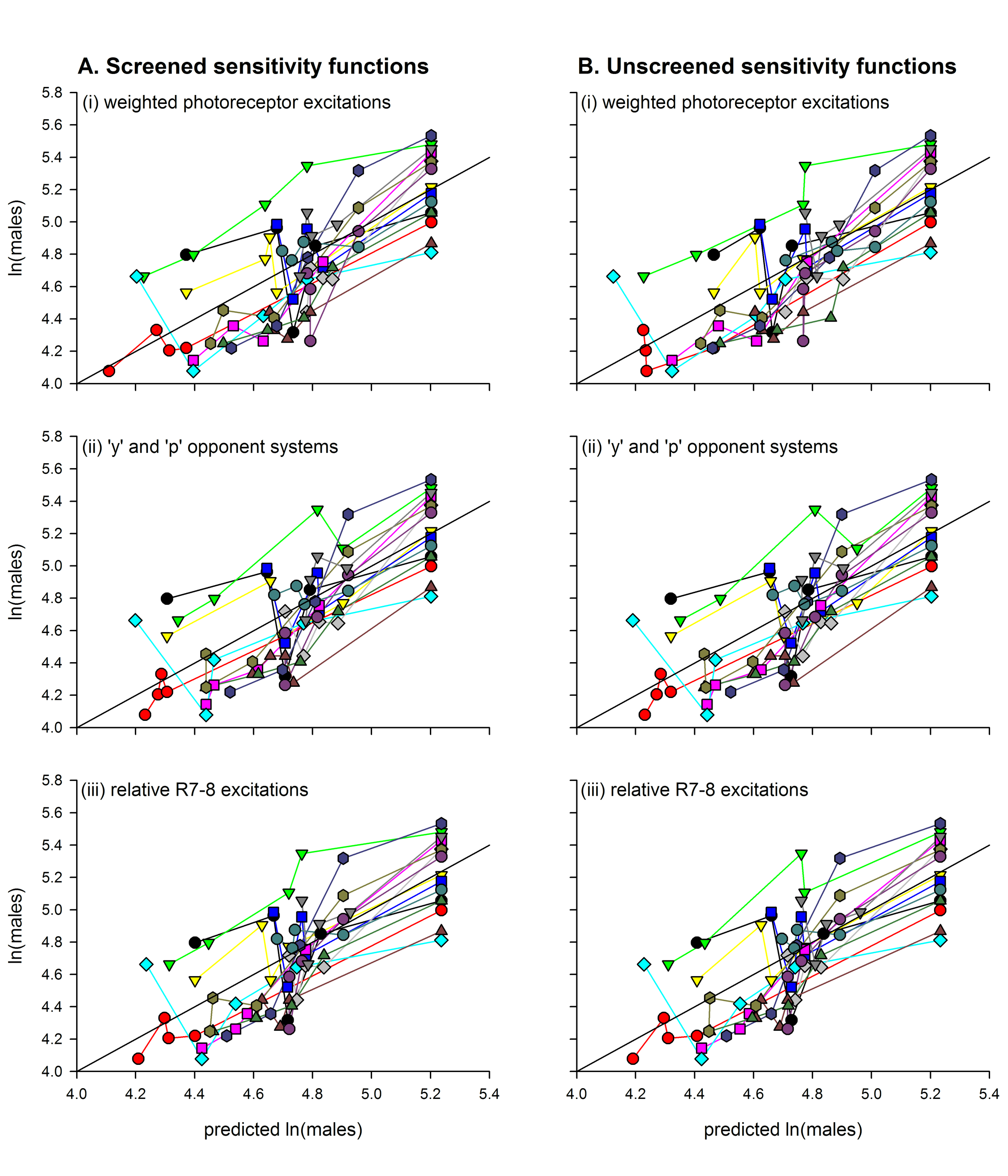

Supplement: S1 Fig — In each plot, natural log-transformed male tsetse catches from a previously published field study [14] are plotted against predicted catches computed using the coefficients generated via GEE analysis, and photoreceptor excitation values calculated from the reflectance spectra of the tiny targets used in the field study. Photoreceptor excitations were calculated using either screened (A), or unscreened (B), sensitivity functions. The models plotted are the best-fitting weighted photoreceptor excitation models (i; Table 3), ‘y’ and ‘p’ opponent system models (ii; Table 4), and relative R7-8 excitation models (iii; Table 5). Data were from 15 separate experiments within the source study, and individual data points from the same experiment have been plotted with the same symbol and colour, and are connected by straight lines. A one-to-one relationship between predicted and actual catches is indicated by the solid, straight line. Because GEE predicts the population-averaged coefficients across experiments, clustering of the data within experiments is evident in the consistently lower or higher catches within that cluster versus predicted values. (TIF) [file pntd.0005448.s004.tif]

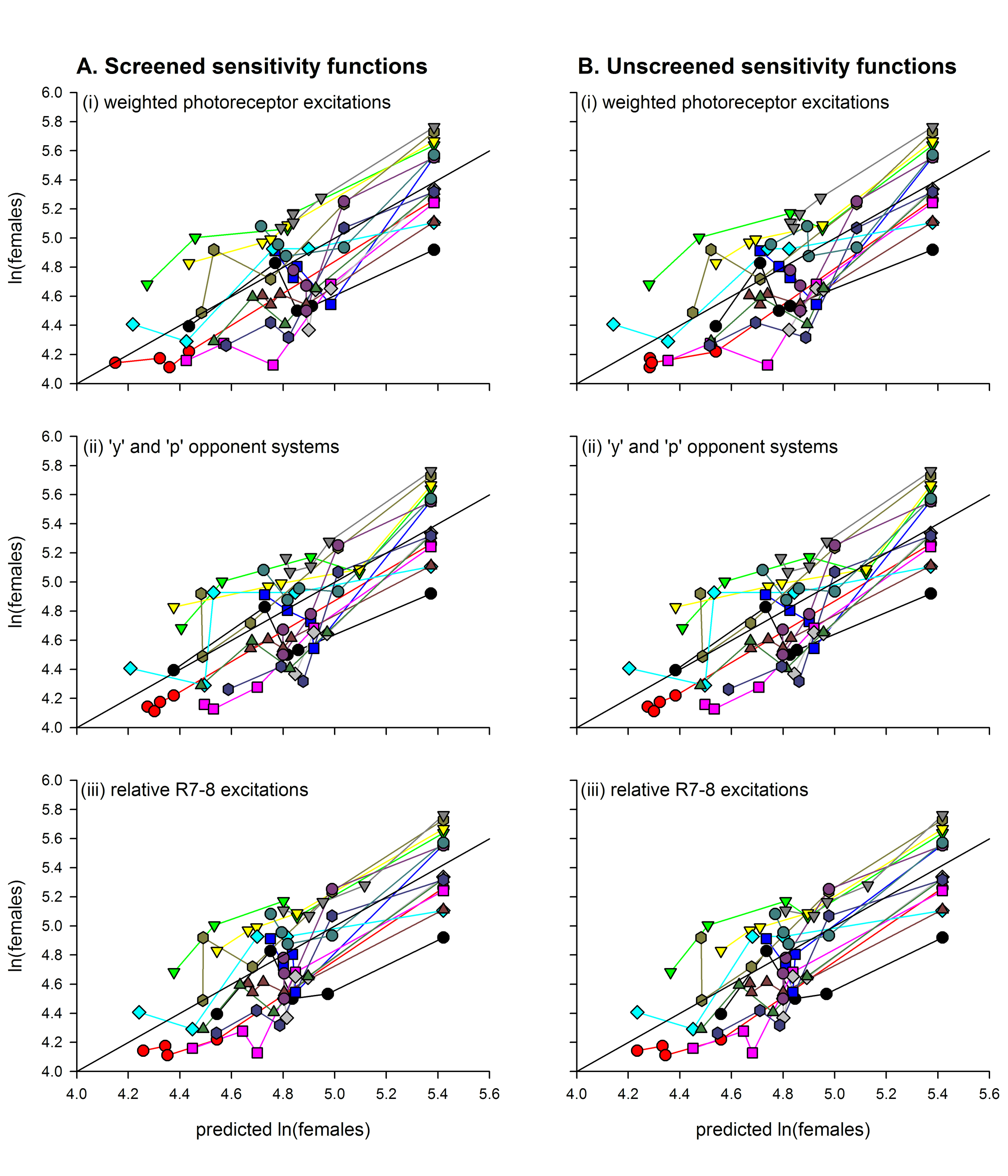

Supplement: S2 Fig — In each plot, natural log-transformed female tsetse catches from a previously published field study [14] are plotted against predicted catches computed using the coefficients generated via GEE analysis, and photoreceptor excitation values calculated from the reflectance spectra of the tiny targets used in the field study. Photoreceptor excitations were calculated using either screened (A), or unscreened (B), sensitivity functions. The models plotted are the best-fitting weighted photoreceptor excitation models (i; Table 3), ‘y’ and ‘p’ opponent system models (ii; Table 4), and relative R7-8 excitation models (iii; Table 5). Data were from 15 separate experiments within the source study, and individual data points from the same experiment have been plotted with the same symbol and colour, and are connected by straight lines. A one-to-one relationship between predicted and actual catches is indicated by the solid, straight line. Because GEE predicts the population-averaged coefficients across experiments, clustering of the data within experiments is evident in the consistently lower or higher catches within that cluster versus predicted values. (TIF) [file pntd.0005448.s005.tif]
