# Supplementary material for: Developing photoreceptor-based models of visual attraction in riverine tsetse, for use in the engineering of more-attractive polyester fabrics for control devices
Source: PLoS Negl Trop Dis. 2017 Mar 17;11(3):e0005448. doi: 10.1371/journal.pntd.0005448 (PMC5371378; doi:10.1371/journal.pntd.0005448)
Supplement: S1 Table — (DOCX) [file pntd.0005448.s006.docx]

**S1 Table: Coefficients for models of tsetse attraction using alternative indices to represent ‘y’ and ‘p’ opponent systems.**

|  | Opponent index | Intercept | | E_R1-6_ | | ‘y’ opponent index | | ‘p’ opponent index | |
| --- | --- | --- | --- | --- | --- | --- | --- | --- | --- |
|  |  | B_0_ | Wald Χ^2^_1_ | B_1_ | Wald Χ^2^_1_ | B_2_ | Wald Χ^2^_1_ | B_3_ | Wald Χ^2^_1_ |
|  |  |  | (p) |  | (p) |  | (p) |  | (p) |
| **A. Males** |  |  |  |  |  |  |  |  |  |
| *(i) Screened* |  |  |  |  |  |  |  |  |  |
|  | E_R7s_-E_R8s_ | 5.167 | 1949.747 | -1.064 | 26.922 | 0.959 | 18.530 | -1.372 | 56.256 |
|  |  |  | **(<0.001)** |  | **(<0.001)** |  | **(<0.001)** |  | **(<0.001)** |
|  | E_R7s_/(E_R7s_+E_R8s_) | 6.088 | 219.275 | -1.111 | 27.123 | 1.653 | 9.407 | -3.318 | 70.857 |
|  |  |  | **(<0.001)** |  | **(<0.001)** |  | **(0.002)** |  | **(<0.001)** |
| *(ii) Unscreened* |  |  |  |  |  |  |  |  |  |
|  | E_R7s_-E_R8s_ | 5.203 | 1993.164 | -1.082 | 27.891 | 1.261 | 16.916 | -1.385 | 55.317 |
|  |  |  | **(<0.001)** |  | **(<0.001)** |  | **(<0.001)** |  | **(<0.001)** |
|  | E_R7s_/(E_R7s_+E_R8s_) | 6.194 | 155.059 | -1.235 | 31.116 | 1.873 | 5.717 | -3.505 | 70.074 |
|  |  |  | **(<0.001)** |  | **(<0.001)** |  | **(0.017)** |  | **(<0.001)** |
| **B. Females** |  |  |  |  |  |  |  |  |  |
| *(i) Screened* |  |  |  |  |  |  |  |  |  |
|  | E_R7s_-E_R8s_ | 5.484 | 2556.621 | -1.374 | 103.864 | 0.789 | 15.302 | -1.691 | 199.972 |
|  |  |  | **(<0.001)** |  | **(<0.001)** |  | **(<0.001)** |  | **(<0.001)** |
|  | E_R7s_/(E_R7s_+E_R8s_) | 7.026 | 974.800 | -1.505 | 164.292 | 1.224 | 11.851 | -4.008 | 223.665 |
|  |  |  | **(<0.001)** |  | **(<0.001)** |  | **(0.001)** |  | **(<0.001)** |
| *(ii) Unscreened* |  |  |  |  |  |  |  |  |  |
|  | E_R7s_-E_R8s_ | 5.515 | 2585.862 | -1.390 | 103.249 | 1.028 | 14.167 | -1.704 | 193.241 |
|  |  |  | **(<0.001)** |  | **(<0.001)** |  | **(<0.001)** |  | **(<0.001)** |
|  | E_R7s_/(E_R7s_+E_R8s_) | 7.089 | 606.198 | -1.588 | 143.910 | 1.403 | 7.435 | -4.145 | 215.408 |
|  |  |  | **(<0.001)** |  | **(<0.001)** |  | **(0.006)** |  | **(<0.001)** |

*Table conventions and interpretation as for Table 3. The relative fits of these models to the data are shown in Table D of S2 Text.*
